# Prenatal Magnesium Sulfate and Functional Connectivity in Offspring at Term-Equivalent Age

**DOI:** 10.1001/jamanetworkopen.2024.13508

**Published:** 2024-05-28

**Authors:** Steven Ufkes, Eleanor Kennedy, Tanya Poppe, Steven P. Miller, Benjamin Thompson, Jessie Guo, Jane E. Harding, Caroline A. Crowther

**Affiliations:** 1 Department of Pediatrics, British Columbia Children’s Hospital, Vancouver, Canada; 2 Department of Pediatrics, University of British Columbia, Vancouver, Canada; 3 Liggins Institute, University of Auckland, Auckland, New Zealand; 4 Centre for the Developing Brain, Department of Biomedical Engineering and Imaging Sciences, King’s College London, London, United Kingdom; 5 School of Optometry and Vision Science, University of Waterloo, Waterloo, Ontario, Canada; 6 Centre for Eye and Vision Research, Hong Kong; 7 Neurosciences and Mental Health, The Hospital for Sick Children Research Institute, Toronto, Ontario, Canada

## Abstract

**Importance:**

Understanding the effect of antenatal magnesium sulfate (MgSO_4_) treatment on functional connectivity will help elucidate the mechanism by which it reduces the risk of cerebral palsy and death.

**Objective:**

To determine whether MgSO_4_ administered to women at risk of imminent preterm birth at a gestational age between 30 and 34 weeks is associated with increased functional connectivity and measures of functional segregation and integration in infants at term-equivalent age, possibly reflecting a protective mechanism of MgSO_4_.

**Design, Setting, and Participants:**

This cohort study was nested within a randomized placebo-controlled trial performed across 24 tertiary maternity hospitals. Participants included infants born to women at risk of imminent preterm birth at a gestational age between 30 and 34 weeks who participated in the MAGENTA (Magnesium Sulphate at 30 to 34 Weeks’ Gestational Age) trial and underwent magnetic resonance imaging (MRI) at term-equivalent age. Ineligibility criteria included illness precluding MRI, congenital or genetic disorders likely to affect brain structure, and living more than 1 hour from the MRI center. One hundred and fourteen of 159 eligible infants were excluded due to incomplete or motion-corrupted MRI. Recruitment occurred between October 22, 2014, and October 25, 2017. Participants were followed up to 2 years of age. Analysis was performed from February 1, 2021, to February 27, 2024. Observers were blind to patient groupings during data collection and processing.

**Exposures:**

Women received 4 g of MgSO_4_ or isotonic sodium chloride solution given intravenously over 30 minutes.

**Main Outcomes and Measures:**

Prior to data collection, it was hypothesized that infants who were exposed to MgSO_4_ would show enhanced functional connectivity compared with infants who were not exposed.

**Results:**

A total of 45 infants were included in the analysis: 24 receiving MgSO_4_ treatment and 21 receiving placebo; 23 (51.1%) were female and 22 (48.9%) were male; and the median gestational age at scan was 40.0 (IQR, 39.1-41.1) weeks. Treatment with MgSO_4_ was associated with greater voxelwise functional connectivity in the temporal and occipital lobes and deep gray matter structures and with significantly greater clustering coefficients (Hedge *g*, 0.47 [95% CI, −0.13 to 1.07]), transitivity (Hedge *g*, 0.51 [95% CI, −0.10 to 1.11]), local efficiency (Hedge *g*, 0.40 [95% CI, −0.20 to 0.99]), and global efficiency (Hedge *g*, 0.31 [95% CI, −0.29 to 0.90]), representing enhanced functional segregation and integration.

**Conclusions and Relevance:**

In this cohort study, infants exposed to MgSO_4_ had greater voxelwise functional connectivity and functional segregation, consistent with increased brain maturation. Enhanced functional connectivity is a possible mechanism by which MgSO_4_ protects against cerebral palsy and death.

## Key Points

**Question:**

Is magnesium sulfate (MgSO_4_) administered to women at risk of imminent preterm birth associated with increased functional connectivity on magnetic resonance imaging (MRI) in infants at term-equivalent age?

**Findings:**

In this cohort study of 45 infants nested within a multicenter randomized, placebo-controlled trial, 24 infants exposed to MgSO_4_ had greater voxelwise functional connectivity on MRI than 21 infants in the placebo group in the temporal and occipital lobes and deep gray matter structures. Exposure to MgSO_4_ was associated with significantly greater measures of functional segregation and integration.

**Meaning:**

These findings suggest that enhanced functional connectivity is a possible mechanism by which MgSO_4_ protects against cerebral palsy and death.

## Teaser

This cohort study investigates whether antenatal magnesium sulfate (MgSO_4_) administration prior to early preterm birth is associated with changes in functional connectivity in the neonatal brain in a cohort of neonates who participated in a multicenter randomized clinical trial.

## Introduction

Preterm birth significantly increases the risk of death and cerebral palsy in infants.^1^ Although medical advances have greatly improved the survival of infants born preterm, the incidence of preterm birth is increasing globally,^2^ and survivors are at increased risk of developmental delay and cerebral palsy and have special educational needs.^3,4^ This places a high financial burden on society and an emotional burden on affected families.^5^

The administration of antenatal magnesium sulfate (MgSO_4_) is an effective treatment for the prevention of cerebral palsy^6^ and is recommended for women at risk of imminent early preterm birth.^7,8,9,10^ Magnesium is important for cellular processes such as protein synthesis^11^ and may reduce the negative downstream effects of brain injury.^12,13^ Antenatal MgSO_4_ reduces the risk of echodensities and echolucencies assessed by cranial ultrasonography^14^ and cerebellar hemorrhage as assessed by magnetic resonance imaging (MRI).^15^ Administration of MgSO_4_ may also enhance early white matter development in very preterm infants,^16^ although this was not evident in infants born at later gestational ages.^17^ However, the neuroprotective effects of MgSO_4_ on other aspects of brain development remain unknown.

The brain is organized as a system of functionally connected networks, typically identified using resting-state functional MRI (rsfMRI). Brain regions with temporally correlated low-frequency fluctuations in the blood oxygen level–dependent (BOLD) signal are said to be functionally connected.^18^ The term *connectome* denotes a map of these functional connections. Network analysis of the connectome quantifies the functional integration and segregation of the brain: segregation reflects the presence of densely interconnected brain regions that are functionally specialized, while integration may reflect the efficiency with which regions communicate.^19,20,21^ The connectome typically has small world organization, meaning that networks are more clustered than random and are balanced in terms of segregation and integration. The topology of the connectome emerges in the second and third trimester of pregnancy,^22^ albeit with immature characteristics. In infants born preterm, aberrant functional connectivity has been linked with neurodevelopmental problems across multiple domains, including motor function and cognition.^18^

The aim of this study was to investigate whether antenatal MgSO_4_ administration prior to early preterm birth alters functional connectivity in the neonatal brain in a cohort of neonates who participated in a multicenter randomized clinical trial. We hypothesized that infants who were exposed to antenatal MgSO_4_ would show enhanced functional connectivity compared with infants who were not exposed.

## Methods

### Participants

This cohort study was nested within the MAGENTA (Magnesium Sulphate at 30 to 34 Weeks’ Gestational Age) trial,^23,24^ a randomized clinical trial performed across 24 tertiary maternity hospitals in New Zealand and Australia. The MAGENTA trial assessed the neuroprotective effects of MgSO_4_ compared with placebo to reduce the incidence of death or cerebral palsy in infants when given to women at risk of imminent preterm birth at a gestational age between 30 and 34 weeks. Participants were infants born to mothers who participated in the MAGENTA trial and who underwent an MRI scan at term-equivalent age as part of the MagNUM (Magnesium Sulfate for Neuroprotection: Understanding Mechanisms) study.^17^ Race and ethnicity data were self-reported by the participant, as required by our ethics approval. Ineligibility criteria for the MagNUM study were infant illness precluding MRI, infant congenital or genetic disorders likely to affect brain structure, and the family living more than 1 hour from the MRI center. Recruitment occurred between October 22, 2014, and October 25, 2017. Participants were followed up to 2 years of age.

The MagNUM study was approved by the New Zealand Northern B Health and Disability Ethics Committees and by the South Australian Human Research Ethics Committee. Caregivers of eligible infants provided written informed consent. This study followed the Strengthening the Reporting of Observational Studies in Epidemiology (STROBE) reporting guideline.

### Treatment Intervention

Women randomized to the MgSO_4_ group were given 4 g of MgSO_4_. Women in the placebo group were given isotonic sodium chloride solution. Treatments were given intravenously over 30 minutes.

### MRI Acquisition

Magnetic resonance imaging was acquired on a 3T device (Skyra; Siemens) with a 32-channel phased-array head coil at The Centre for Advanced MRI, Auckland, New Zealand, and at the South Australia Medical Research Institute, Adelaide, and on another similar 3T device (HDxt; General Electric) at Hagley Radiology, St Georges Hospital, Christchurch, New Zealand. Infants were scanned without sedation during natural sleep. The MRI protocol included anatomical (T1- and T2-weighted), resting state, and diffusion scans. Diffusion imaging findings have been reported previously.^17^

In the Skyra device, scans included a T1-weighted magnetization-prepared rapid acquisition gradient-echo pulse sequence (repetition time [TR], 2000 milliseconds; echo time [TE], 2.83 milliseconds; inversion time [TI], 900 milliseconds; 1 mm^3^ voxels; and 220-mm field of view [FOV]) and rsfMRI acquired with a gradient-echo, echo-planar image (EPI) sequence (TR, 3000 milliseconds; TE, 27.0 milliseconds; 2-mm^3^ voxels; flip angle, 90°; and 120 volumes). In the HDxt device, scans included a T1-weighted spoiled gradient recalled acquisition in steady state (TE, 2.504 milliseconds; TR, 5.988 milliseconds; flip angle, 15°; TI, 400 milliseconds; 1-mm^3^ voxels; and FOV, 220 × 220 mm) and rsfMRI also acquired with a gradient-echo, EPI sequence (TR, 3000 milliseconds; TE, 27.0 milliseconds; 2-mm^3^ voxels; flip angle, 90°; and 120 volumes).

### MRI Preprocessing

All MRI preprocessing and scan exclusions were performed while blind to treatment allocation. After excluding scans of infants whose fMRI or T1-weighted volumes were incomplete or severely motion corrupted based on visual inspection, the T1-weighted images were used to construct an age-appropriate anatomical template using Advanced Normalization Tools (ANTs).^25,26^ We obtained a brain mask for the template by warping an existing neonatal atlas^27^ to our template using ANTs.

### fMRI Preprocessing

Preprocessing of fMRI data was performed using FSL,^28^ including intervolume motion correction,^29^ spatial smoothing using a Gaussian kernel with a full-width half-maximum of 5 mm, and high-pass temporal filtering to remove low-frequency (<0.01 Hz) fluctuations. We removed volumes at the start and end of each series for which the relative motion between consecutive frames exceeded 0.25 mm. We excluded scans not having at least 5 continuous minutes in which the relative root-mean-squared displacement between consecutive frames did not exceed 1 mm. After volume removal, scan lengths ranged from 100 to 120 frames (5-6 minutes).

To align fMRI data across participants, each participant’s fMRI image was aligned to their anatomical image, then warped to the anatomical template. Nonbrain regions were then removed using the template brain mask.

We performed independent component analysis denoising to reduce fMRI signal contributions from head motion, cerebral blood flow, cerebrospinal fluid, and white matter using MELODIC (multivariate exploratory linear optimized decomposition into independent components)^30^ and following published guidelines.^31,32^ We delineated 92 anatomical regions of interest, comprising left and right components of 45 regions defined in the Automated Anatomical Labeling map,^33^ and the cerebellar hemispheres by warping 2 neonatal atlases^34,35^ to our template and combining labels.

### Functional Connectivity

We defined functional connectivity as the absolute value of the Pearson correlation coefficient between 2 BOLD signals and assessed connectivity at 2 scales: first at the voxel level comparing signals between pairs of voxels, then at the regional level comparing the mean BOLD signals between pairs of anatomical regions. We calculated connectivity between pairs of voxels lying within the 92 anatomical regions. The connectivities between voxels lying within 10 mm of each other were zeroed to mitigate contributions from shared signals.^36^ At each voxel, we calculated the voxel mean connectivity as the mean of the connectivities between the voxel and all others.

We examined the connectivity between each pair of anatomical regions. At each region, we also calculated the region mean connectivity as the mean of the connectivities between the region and all others.

### Functional Network Analysis

#### Network Construction

To assess functional segregation and integration, we constructed weighted functional networks for each participant using the 92 anatomical regions as nodes. To construct edges, we used a proportional thresholding approach in which the ratio of actual connections to possible connections was set to a fixed density for all participants. The weights of the edges connecting 2 nodes were initially set to the absolute value of the correlation coefficient. The lowest-weight edges were then set to zero, denoting nonconnections, until the specified density was reached.

Because network properties vary with the density^21^ and there is no established way to select a single density threshold, we constructed networks over a range of densities between 0.10 and 0.35 in steps of 0.01.^37^ As various definitions of functional connectivity exist, we constructed an alternative set of networks, with edge weights defined as the Fisher *z*-transformed correlation coefficients after zeroing negative correlations.

#### Network Metrics

We compared global and nodal network properties between the treatment and placebo groups. All measures were computed using the Brain Connectivity Toolbox for Python.^21^ We hypothesized that MgSO_4_ treatment would be associated with greater measures of functional segregation, integration, and small-worldness.

##### Functional Segregation and Integration

We assessed 4 measures of functional segregation: clustering coefficient^38^ at the network and node levels, which reflects the presence of densely interconnected subsystems of nodes; transitivity, which is the collectively normalized index of clustering coefficient; local efficiency at the network and node levels, which measures the efficiency of information exchange between a node’s neighbors when the node is removed; and modularity, which indicates how well the whole-brain network can be segregated into distinct subsystems. We also assessed 2 measures of functional integration: characteristic path length, which is the mean of the shortest path lengths across all pairs of nodes; and global efficiency, defined as the mean of inverse shortest path lengths. Greater integration corresponds to shorter characteristic path length and greater global efficiency.

##### Small-Worldness

We calculated small-worldness for each network,^39^ which is the ratio of the clustering coefficient to the characteristic path length after normalizing each by its value in a random network. We normalized by dividing each metric by its average value across 100 random networks with the same size and edge weight distribution.

#### Area Under the Curve

To ensure that findings were not tied to a specific density threshold, we used an area under the curve method.^40^ Network metrics were computed at each threshold, and the following value was analyzed:
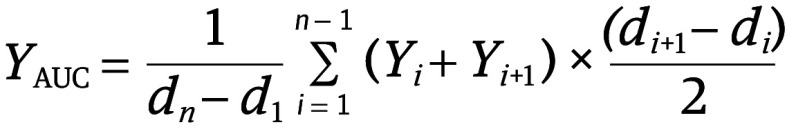
where *Y_i_* is the metric value at the *i*^th^ network density, *d_i_*. This represents the mean of the metric over the range of densities, approximated using the trapezoidal rule. To further validate our findings, we assessed metrics at each density separately.

### Statistical Analysis

Data were analyzed from February 1, 2021, to February 27, 2024. All statistical tests adjusted for the interaction between sex and gestational age at birth and for the MRI scan site, as described previously.^17^ We performed additional sensitivity analysis including only data from the largest MRI site. We measured effect size using Hedge *g* statistic.

To compare voxel mean connectivity between treatment groups, we performed 2-sided nonparametric permutation tests with 10 000 permutations of participants using FSL permutation analysis of linear models (PALM) with threshold-free cluster enhancement,^41^ correcting for multiple comparisons across voxels. To compare regional mean connectivity between treatment groups and nodal and network-wide network properties between treatment groups, we performed 2-sided nonparametric permutation tests with 100 000 permutations of participants, correcting for multiple comparisons across regions using Benjamini-Hochberg false discovery rate (FDR) correction.^42^ Connectivities between pairs of regions were assessed by the same method with 10 000 permutations of participants, correcting for multiple comparisons across all 4186 region pairs. These permutation tests were performed using the R package permuco, version 4.0.3 (R Project for Statistical Computing), accounting for covariates using the Freedman-Lane method.^43^ Two-sided *P* < .05 indicated statistical significance.

## Results

### Participants

A total of 45 infants were included in the analysis (Table 1): 24 in the MgSO_4_ group, and 21 in the placebo group; of these, 23 (51.1%) were female and 22 (48.9%) were male. The median gestational age at scan was 40.0 (IQR, 39.1-41.1) weeks, and the median gestational age at birth was 32.1 (IQR, 31.3-33.0) weeks. Among the 40 mothers included, 9 (22.5%) were Asian, 5 (12.5%) were Maori, 4 (10.0%) were Polynesian, 14 (35.0%) were White, and 8 (20.0%) were of other race or ethnicity (including African and Middle Eastern). Maternal demographic characteristics were similar between the treatment groups. Infant characteristics, including gestational age at scan and multiple births, did not differ significantly between treatment groups.

**Table 1.** Characteristics of Mothers and Infants^a^

Characteristic	Treatment group		*P* value
	MgSO_4_	Placebo	
**Mothers**			
No. of participants	22	18	NA
Age, mean (SD), y	29.4 (6.6)	31.7 (5.7)	.24
Parity	13 (59.1)	8 (44.4)	.53
Race and ethnicity, No. (%)			
Asian	3 (13.6)	6 (33.3)	.60
Maori	3 (13.6)	2 (11.1)	
Polynesian	3 (13.6)	1 (5.6)	
White	9 (40.9)	5 (27.8)	
Other^b^	4 (18.2)	4 (22.2)	
BMI, mean (SD)	29.4 (7.7)	26.3 (5.8)	.17
GA at entry, median (IQR), wk	32.3 (31.4-33.0)	32.0 (30.9-32.9)	.33
Main risk for preterm birth			
Antepartum hemorrhage	4 (18.2)	0	.19
PPROM	7 (31.8)	4 (22.2)	
Preterm labor	7 (31.8)	11 (61.1)	
Preeclampsia	5 (22.7)	3 (16.7)	
Fetal compromise	2 (9.1)	2 (11.1)	
Other	6 (27.3)	1 (5.6)	
Received allocated treatment	22 (100)	18 (100)	NA
**Infants**			
No. of participants	24	21	NA
GA at birth, median (IQR), wk	32.3 (31.7-33.1)	32.1 (31.0-32.6)	.20
Singleton	19 (79.2)	14 (66.7)	.64
Twin 1	3 (12.5)	4 (19.0)	
Twin 2	2 (8.3)	3 (14.3)	
MRI site			
Auckland CAMRI	18 (75.0)	14 (66.7)	.73
Christchurch MRI	5 (20.8)	5 (23.8)	
Adelaide SAHMRI	1 (4.2)	2 (9.5)	
PMA at MRI, median (IQR), wk	40.0 (39.1-41.2)	39.9 (39.4-41.1)	.92
Birth weight, mean (SD), g	1925 (583)	1693 (389)	.13
Birth weight *z* score, mean (SD)	0.5 (1.3)	−0.0 (0.9)	.12
Bronchopulmonary dysplasia	2 (8.3)	2 (9.5)	.89
Necrotizing enterocolitis	0	0	NA
Full breast milk feeding at discharge	12 (50.0)	13 (61.9)	.67
Sex			
Female	14 (58.3)	9 (42.9)	.30
Male	10 (41.7)	12 (57.1)	
Relative RMS displacement between consecutive frames, mean (SD), mm	0.066 (0.027)	0.061 (0.023)	.45

Abbreviations: BMI, body mass index (calculated as weight in kilograms divided by height in meters squared); CAMRI, Centre for Advanced MRI; GA, gestational age; MgSO_4_, magnesium sulfate; MRI, magnetic resonance imaging; NA, not applicable; PMA, postmenstrual age; PPROM, preterm prelabor rupture of the membranes; RMS, root-mean-squared; SAHMRI, South Australia Medical Research Institute.

^a^ Unless otherwise indicated, data are expressed as No. (%) of participants.

^b^ Includes African and Middle Eastern.

**Figure 1.**
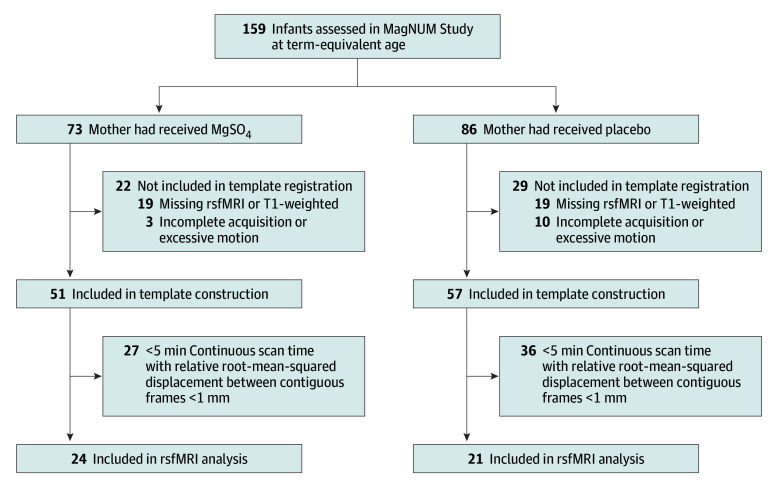
Flow Diagram of Participants Included in the Resting-State Functional Magnetic Resonance Imaging (rsfMRI) Analysis MagNUM indicates Magnesium Sulfate for Neuroprotection: Understanding Mechanisms; MgSO_4_, magnesium sulfate.

A total of 159 infants had MRI scans as part of the MagNUM Study: 73 in the MgSO_4_ group and 86 in the placebo group. Thirty-eight infants had no rsfMRI or T1-weighted images, and 13 had excessive motion assessed visually. An additional 63 failed the 5-minute motion criterion (Figure 1). Maternal and neonatal characteristics, including treatment allocation, were similar between included and excluded infants (eTable 1 in Supplement 1).

### Voxelwise Functional Connectivity

**Figure 2.**
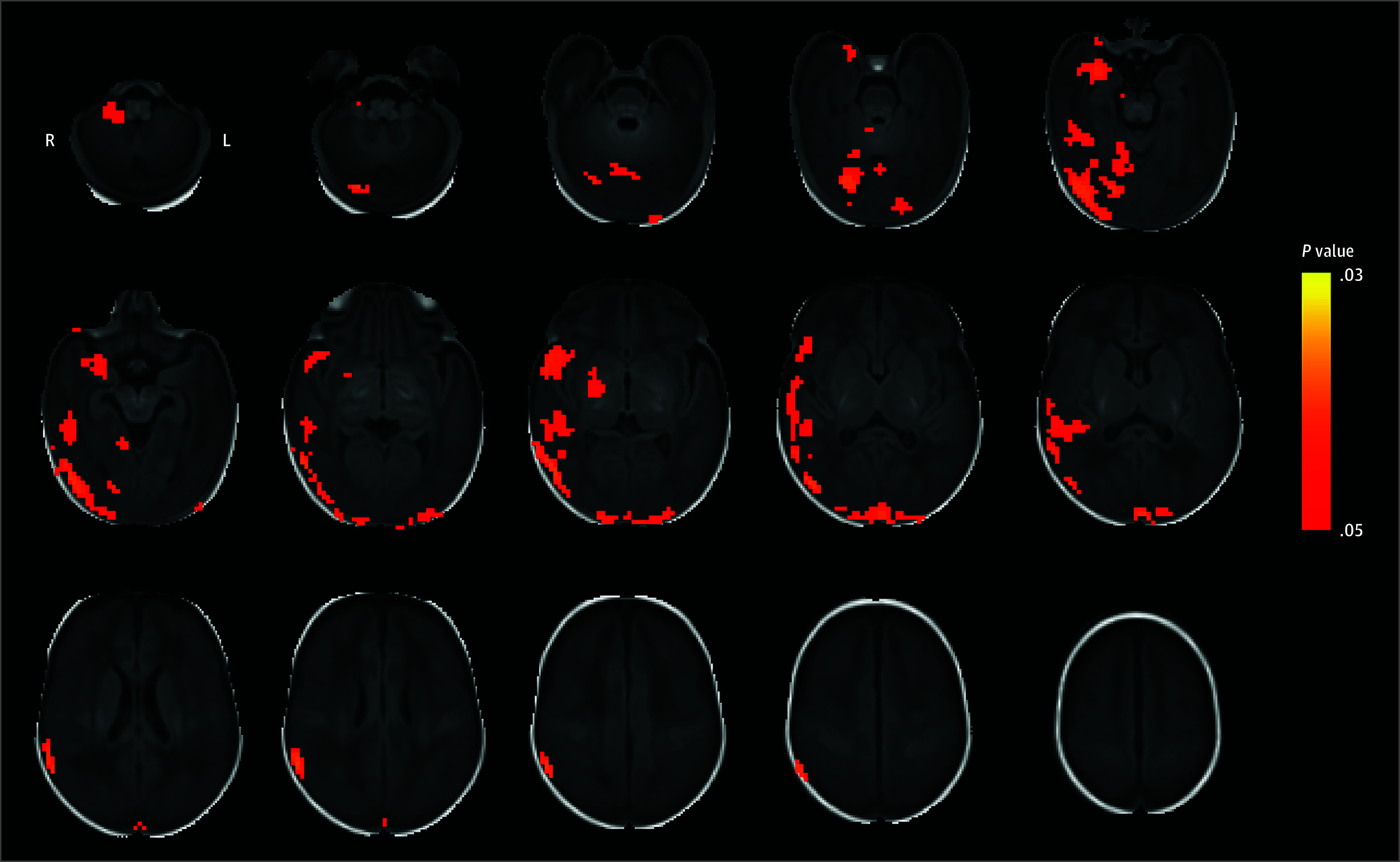
Comparison of Voxel Mean Connectivity Between Treatment Groups Magnesium sulfate (MgSO_4_) was associated with a widespread increase in voxel mean connectivity. Red-yellow voxels represent *P* values for the test that voxel mean connectivity differs between the MgSO_4_ and placebo groups, shown where *P* < .05, corrected for multiple comparisons. L indicates left; R, right.

Treatment with MgSO_4_ was associated with greater voxel mean connectivity in the right temporal lobe (association in 7080 mm^3^), occipital lobe (association in 1832 mm^3^), deep gray matter structures in the right hemisphere (association in 760 mm^3^), and the right cerebellar hemisphere (association in 392 mm^3^) (Figure 2 and eTable 2 in Supplement 1). The regions with the greatest volumes of significant voxels were the right middle and superior temporal gyri. When including only data from the largest MRI site (n = 32), the direction was unchanged in all voxels that were significant in the primary analysis, although most did not remain significant.

### Regional Functional Connectivity

Regional mean connectivity was positively associated with MgSO_4_ treatment in all 92 regions (eFigure 1 and eTable 5 in Supplement 1), but none of the associations remained (*P* < .05) after FDR correction. Similarly, connectivity between most region pairs was positively associated with MgSO_4_ treatment, but these associations did not remain (*P* < .05) after FDR correction across all 4186 pairs. To illustrate which connections exhibited the greatest between-group differences, we indicate the region pairs whose *t* values were in the highest 5% among all pairs (eFigure 1 in Supplement 1).

### Network Metrics

Treatment with MgSO_4_ was associated with significantly enhanced functional segregation, as indicated by greater clustering coefficients, transitivity, and local efficiency (Table 2); however, modularity differed little between treatment groups. These associations held when each density was assessed individually (eFigure 2 in Supplement 1); they were also consistent with those in the alternatively defined networks (eTable 3 in Supplement 1) and when only data from the largest site (n = 32) were included (eTable 4 in Supplement 1).

**Table 2.** Comparison of Global Network Metrics Between Treatment Groups

Metric	Treatment, mean (SD) AUC		Effect size, Hedge *g* statistic (95% CI)	*P* value
	Placebo	MgSO_4_		
Characteristic path length	3.04 (0.41)	2.92 (0.39)	−0.30 (−0.89 to 0.30)	.05
Global efficiency	0.34 (0.05)	0.35 (0.05)	0.31 (−0.29 to 0.90)	.047
Clustering coefficient	0.38 (0.08)	0.42 (0.09)	0.47 (−0.13 to 1.07)	.01
Transitivity	0.38 (0.09)	0.43 (0.10)	0.51 (−0.10 to 1.11)	.02
Local efficiency	0.54 (0.09)	0.57 (0.10)	0.40 (−0.20 to 0.99)	.02
Modularity	0.29 (0.05)	0.30 (0.07)	0.16 (−0.44 to 0.75)	.71
Small-worldness	1.43 (0.24)	1.45 (0.28)	0.08 (−0.51 to 0.68)	.85

Abbreviations: AUC, area under the curve; MgSO_4_, magnesium sulfate.

At the regional level, MgSO_4_ treatment was associated with an increase in the clustering coefficient in most of the nodes (eTable 6 in Supplement 1). After FDR correction, only the association in the right middle temporal gyrus remained significant (Hedge *g*, 1.07 [95% CI, 0.44-1.70]; *P* = .003). Similarly, MgSO_4_ treatment was associated with an increase in local efficiency in most regions (eTable 7 in Supplement 1), but only the association in the right middle temporal gyrus remained (Hedge *g*, 0.87 [95% CI, 0.25-1.49]; *P* = .02) after FDR correction. Findings were similar in the alternatively defined networks: MgSO_4_ treatment was associated with significantly increased clustering coefficient (Hedge *g*, 1.03 [95% CI, 0.40-1.66]; *P* = .003) and local efficiency (Hedge *g*, 0.91 [95% CI, 0.28-1.53]; *P* = .006) only in the right middle temporal gyrus after FDR correction. When only data from the largest site were included, the directions of clustering coefficient (Hedge *g*, 1.01 [95% CI, 0.26-1.77]; *P* = .003) and local efficiency (Hedge *g*, 0.77 [95% CI, 0.03-1.50]; *P* = .10) were unchanged in the right middle temporal gyrus.

Treatment with MgSO_4_ was associated with significantly greater clustering coefficients (Hedge *g*, 0.47 [95% CI, −0.13 to 1.07]), transitivity (Hedge *g*, 0.51 [95% CI, −0.10 to 1.11]), local efficiency (Hedge *g*, 0.40 [95% CI, −0.20 to 0.99]), global efficiency (Hedge *g*, 0.31 [95% CI, −0.29 to 0.90]) and a shorter characteristic path length (Hedge *g*, −0.30 [95% CI, −0.89 to 0.30]) (Table 2). These findings were consistent with those in the alternatively defined networks (eTable 3 in Supplement 1) and when only data from the largest site were included (eTable 4 in Supplement 1). No substantial difference in small-worldness was found between treatment groups in either set of networks (Table 2 and eTable 3 in Supplement 1).

## Discussion

In this nested multicenter cohort study, stronger voxelwise connectivity and greater functional segregation was observed in infants exposed to antenatal MgSO_4_. During the third trimester, functional connectivity and measures of segregation increase,^36^ so these findings may reflect enhanced brain maturation in the MgSO_4_ group. This is consistent with evidence of reduced risk of brain anomalies^14,15^ and suggests that exposure to antenatal MgSO_4_ may lead to resistance to brain injury in part by accelerating early-life brain maturation.

Preterm infants have reportedly weaker intrinsic brain activity when compared with full-term infants at term-equivalent age^44,45^ and in adolescence.^46^ We found that preterm infants exposed to antenatal MgSO_4_ had stronger voxelwise connectivity in the temporal and occipital lobes and deep gray matter structures. Connectivity in regions important for motor function, such as the precentral gyrus and thalamus, was stronger in the MgSO_4_ treatment group, consistent with the role of MgSO_4_ in preventing cerebral palsy. In addition, we observed stronger connectivity in visual, auditory, and olfactory regions, such as the occipital lobes, superior temporal gyrus, and parahippocampal gyrus. Primary sensory networks mature earlier than higher-order association networks^18^ and show the greatest increases in functional connectivity strength during the third trimester,^36^ so these findings could also suggest enhanced maturation in the MgSO_4_ group. The regional connectivity findings are consistent with this, although they were not statistically significant after FDR correction.

Network construction may influence group comparisons,^21^ so we tested our findings across a range of density thresholds and found consistent results. An investigation into network development in preterm and term infants demonstrated increasing network segregation between 31 and 42 weeks of postmenstrual age, and preterm infants (gestational age <32 weeks) have shown reduced network segregation relative to full-term infants at term-equivalent age.^47^ Our network findings may therefore imply enhanced brain maturation in the MgSO_4_ group.

The positive associations we found between MgSO_4_ and functional connectivity contrast our diffusion imaging findings, which suggested that MgSO_4_ may be related to delayed white matter maturation inferred from fractional anisotropy.^17^ It is possible that the enhanced connectivity reflects a compensatory mechanism to overcome slower white matter maturation, as proposed for patients with chronic stroke.^48^ Alternatively, because rsfMRI measures rely on the BOLD signal, they may be more sensitive to beneficial hemodynamic effects of MgSO_4_, such as increased cerebral perfusion and stabilization of neonatal blood pressure variability.^49,50^

### Strengths and Limitations

We applied stringent criteria for motion correction to improve the accuracy of activation maps.^51^ Furthermore, fMRI series were acquired later in the MRI session when the infants were often unsettled. This resulted in a small sample size, which, although similar in size to previous studies on functional connectivity in preterm infants,^36,44,52,53^ would have reduced statistical power to detect subtle differences. However, clinical characteristics were similar between included and excluded infants, suggesting that included infants were representative of the MagNUM cohort. The use of data from a randomized clinical trial is a considerable strength of this study, and the participants included in the analysis were well balanced for baseline characteristics.

The MRI scans were acquired at 3 different sites, each with a different scanner. While MRI protocols were matched as closely as possible and statistical models accounted for site, this may still have been a confounder of our findings. However, our primary findings were bolstered by their consistency with sensitivity analyses including only data from the largest site. Last, despite measures to minimize and correct for confounders and outcome modifiers, certain variables were unexamined or uncollected, including the presence of punctuate white matter injury or cerebellar microhemorrhages.

## Conclusions

In this cohort study, infants exposed to antenatal MgSO_4_ showed potentially enhanced brain connectivity reflected in stronger voxelwise functional connectivity, segregation, and integration on fMRI at term-equivalent age. These findings suggest a possible mechanism by which administration of antenatal MgSO_4_ when birth is imminent at a gestational age of 30 to 34 weeks may be neuroprotective for the preterm infant brain. Future studies exploring the association between these MRI findings and neurodevelopmental outcomes will assist with their interpretation.

## Notes

Supplement 1. **eTable 1.** Characteristics of Mothers and Infants Included in and Excluded From the Resting-State fMRI Analyses **eTable 2.** Volumes of Voxels in Which a Significant (*P* < .05) Association Between Voxel Mean Connectivity and Magnesium Sulfate (MgSO_4_) Was Detected **eFigure 1.** Region Mean Connectivity and Connectivity Between Regions **eTable 3.** Comparison of Global Network Metrics Between Treatment Groups in Alternate Set of Networks **eTable 4.** Comparison of Global Network Metrics Between Treatment Groups Within the Largest MRI Site (n = 32) **eFigure 2.** Network Metrics at Each Density Threshold **eTable 5.** Functional Connectivity at Each Node **eTable 6.** Clustering Coefficient AUC at Each Node **eTable 7.** Local Efficiency AUC at Each Node

Supplement 2.
Data Sharing Statement

## References

[1] Saigal S, Doyle LW. An overview of mortality and sequelae of preterm birth from infancy to adulthood. Lancet. 2008;371(9608):261-269. doi:10.1016/S0140-6736(08)60136-1 18207020

[2] Walani SR. Global burden of preterm birth. Int J Gynaecol Obstet. 2020;150(1):31-33. doi:10.1002/ijgo.13195 32524596

[3] Blencowe H, Lee ACC, Cousens S, . Preterm birth-associated neurodevelopmental impairment estimates at regional and global levels for 2010. Pediatr Res. 2013;74(suppl 1):17-34. doi:10.1038/pr.2013.204 24366461 PMC3873710

[4] Blencowe H, Cousens S, Oestergaard MZ, . National, regional, and worldwide estimates of preterm birth rates in the year 2010 with time trends since 1990 for selected countries: a systematic analysis and implications. Lancet. 2012;379(9832):2162-2172. doi:10.1016/S0140-6736(12)60820-4 22682464

[5] Khan KA, Petrou S, Dritsaki M, . Economic costs associated with moderate and late preterm birth: a prospective population-based study. BJOG. 2015;122(11):1495-1505. doi:10.1111/1471-0528.13515 26219352

[6] Crowther CA, Middleton PF, Voysey M, ; AMICABLE Group. Assessing the neuroprotective benefits for babies of antenatal magnesium sulphate: an individual participant data meta-analysis. PLoS Med. 2017;14(10):e1002398. doi:10.1371/journal.pmed.1002398 28976987 PMC5627896

[7] Magee L, Sawchuck D, Synnes A, von Dadelszen P; Magnesium Sulphate for Fetal Neuroprotection Consensus Committee; Maternal Fetal Medicine Committee. SOGC clinical practice guideline: magnesium sulphate for fetal neuroprotection. J Obstet Gynaecol Can. 2011;33(5):516-529. doi:10.1016/S1701-2163(16)34886-1 21639972

[8] Committee Opinion No. 455: magnesium sulfate before anticipated preterm birth for neuroprotection. Obstet Gynecol. 2010;115(3):669-671. doi:10.1097/AOG.0b013e3181d4ffa5 20177305

[9] National Institute for Care and Excellence. Quality statement 6: magnesium sulfate for women between 24+0 and 29+6 weeks of pregnancy. Updated August 2, 2019. Accessed April 17, 2024. https://www.nice.org.uk/guidance/qs135/chapter/Quality-statement-6-Magnesium-sulfate-for-women-between-240-and-296-weeks-of-pregnancy

[10] The Antenatal Magnesium Sulphate For Neuroprotection Guideline Development Panel. Antenatal magnesium sulphate prior to preterm birth for neuroprotection of the fetus, infant and child: national clinical practice guidelines. 2010. Accessed April 17, 2024. https://ontrack.perinatalsociety.org.nz/wp-content/uploads/2020/04/Antenatal-magnesium-sulphate-prior-to-preterm-birth-for-neuroprotection-of-the-fetus-infant-child-National-clinical-practice-guidelines.pdf

[11] Mildvan AS. Role of magnesium and other divalent cations in ATP-utilizing enzymes. Magnesium. 1987;6(1):28-33. 3029516

[12] Nakagawa M, Oono H, Nishio A. Enhanced production of IL-1β and IL-6 following endotoxin challenge in rats with dietary magnesium deficiency. J Vet Med Sci. 2001;63(4):467-469. doi:10.1292/jvms.63.467 11346186

[13] Nowak L, Bregestovski P, Ascher P, Herbet A, Prochiantz A. Magnesium gates glutamate-activated channels in mouse central neurones. Nature. 1984;307(5950):462-465. doi:10.1038/307462a0 6320006

[14] Hirtz DG, Weiner SJ, Bulas D, ; Eunice Kennedy Shriver National Institute of Child Health and Human Development Maternal-Fetal Medicine Units Network. Antenatal magnesium and cerebral palsy in preterm infants. J Pediatr. 2015;167(4):834-839.e3. doi:10.1016/j.jpeds.2015.06.067 26254839 PMC4587284

[15] Gano D, Ho ML, Partridge JC, . Antenatal exposure to magnesium sulfate is associated with reduced cerebellar hemorrhage in preterm newborns. J Pediatr. 2016;178:68-74. doi:10.1016/j.jpeds.2016.06.053 27453378 PMC5085851

[16] Anblagan D, Bastin ME, Sparrow S, . Tract shape modeling detects changes associated with preterm birth and neuroprotective treatment effects. Neuroimage Clin. 2015;8:51-58. doi:10.1016/j.nicl.2015.03.021 26106527 PMC4473726

[17] Poppe T, Thompson B, Boardman JP, ; MagNUM Study Group. Effect of antenatal magnesium sulphate on MRI biomarkers of white matter development at term equivalent age: the MagNUM Study. EBioMedicine. 2022;78:103923. doi:10.1016/j.ebiom.2022.103923 35331677 PMC9043972

[18] Rogers CE, Lean RE, Wheelock MD, Smyser CD. Aberrant structural and functional connectivity and neurodevelopmental impairment in preterm children. J Neurodev Disord. 2018;10(1):38. doi:10.1186/s11689-018-9253-x 30541449 PMC6291944

[19] Kim DJ, Davis EP, Sandman CA, . Longer gestation is associated with more efficient brain networks in preadolescent children. Neuroimage. 2014;100:619-627. doi:10.1016/j.neuroimage.2014.06.048 24983711 PMC4138264

[20] Smyser CD, Wheelock MD, Limbrick DD Jr, Neil JJ. Neonatal brain injury and aberrant connectivity. Neuroimage. 2019;185:609-623. doi:10.1016/j.neuroimage.2018.07.057 30059733 PMC6289815

[21] Rubinov M, Sporns O. Complex network measures of brain connectivity: uses and interpretations. Neuroimage. 2010;52(3):1059-1069. doi:10.1016/j.neuroimage.2009.10.003 19819337

[22] Turk E, van den Heuvel MI, Benders MJ, . Functional connectome of the fetal brain. J Neurosci. 2019;39(49):9716-9724. doi:10.1523/JNEUROSCI.2891-18.2019 31685648 PMC6891066

[23] Crowther CA, Middleton PF, Wilkinson D, Ashwood P, Haslam R; MAGENTA Study Group. Magnesium sulphate at 30 to 34 weeks’ gestational age: neuroprotection trial (MAGENTA)—study protocol. BMC Pregnancy Childbirth. 2013;13(1):91. doi:10.1186/1471-2393-13-91 23570677 PMC3636106

[24] Crowther CA, Ashwood P, Middleton PF, McPhee A, Tran T, Harding JE; MAGENTA Study Group. Prenatal intravenous magnesium at 30-34 weeks’ gestation and neurodevelopmental outcomes in offspring: the MAGENTA randomized clinical trial. JAMA. 2023;330(7):603-614. doi:10.1001/jama.2023.12357 37581672 PMC10427942

[25] Avants BB, Tustison NJ, Song G, Cook PA, Klein A, Gee JC. A reproducible evaluation of ANTs similarity metric performance in brain image registration. Neuroimage. 2011;54(3):2033-2044. doi:10.1016/j.neuroimage.2010.09.025 20851191 PMC3065962

[26] Avants BB, Epstein CL, Grossman M, Gee JC. Symmetric diffeomorphic image registration with cross-correlation: evaluating automated labeling of elderly and neurodegenerative brain. Med Image Anal. 2008;12(1):26-41. doi:10.1016/j.media.2007.06.004 17659998 PMC2276735

[27] Serag A, Aljabar P, Ball G, . Construction of a consistent high-definition spatio-temporal atlas of the developing brain using adaptive kernel regression. Neuroimage. 2012;59(3):2255-2265. doi:10.1016/j.neuroimage.2011.09.062 21985910

[28] Jenkinson M, Beckmann CF, Behrens TEJJ, Woolrich MW, Smith SM. FSL. Neuroimage. 2012;62(2):782-790. doi:10.1016/j.neuroimage.2011.09.015 21979382

[29] Jenkinson M, Bannister P, Brady M, Smith S. Improved optimization for the robust and accurate linear registration and motion correction of brain images. Neuroimage. 2002;17(2):825-841. doi:10.1006/nimg.2002.1132 12377157

[30] Beckmann CF, Smith SM. Probabilistic independent component analysis for functional magnetic resonance imaging. IEEE Trans Med Imaging. 2004;23(2):137-152. doi:10.1109/TMI.2003.822821 14964560

[31] Kelly RE Jr, Alexopoulos GS, Wang Z, . Visual inspection of independent components: defining a procedure for artifact removal from fMRI data. J Neurosci Methods. 2010;189(2):233-245. doi:10.1016/j.jneumeth.2010.03.028 20381530 PMC3299198

[32] Griffanti L, Douaud G, Bijsterbosch J, . Hand classification of fMRI ICA noise components. Neuroimage. 2017;154:188-205. doi:10.1016/j.neuroimage.2016.12.036 27989777 PMC5489418

[33] Tzourio-Mazoyer N, Landeau B, Papathanassiou D, . Automated anatomical labeling of activations in SPM using a macroscopic anatomical parcellation of the MNI MRI single-subject brain. Neuroimage. 2002;15(1):273-289. doi:10.1006/nimg.2001.0978 11771995

[34] Shi F, Yap PT, Wu G, . Infant brain atlases from neonates to 1- and 2-year-olds. PLoS One. 2011;6(4):e18746. doi:10.1371/journal.pone.0018746 21533194 PMC3077403

[35] Oishi K, Mori S, Donohue PK, . Multi-contrast human neonatal brain atlas: application to normal neonate development analysis. Neuroimage. 2011;56(1):8-20. doi:10.1016/j.neuroimage.2011.01.051 21276861 PMC3066278

[36] Cao M, He Y, Dai Z, . Early development of functional network segregation revealed by connectomic analysis of the preterm human brain. Cereb Cortex. 2017;27(3):1949-1963. doi:10.1093/cercor/bhw038 26941380 PMC6059235

[37] Watts DJ, Strogatz SH. Collective dynamics of “small-world” networks. Nature. 1998;393(6684):440-442. doi:10.1038/30918 9623998

[38] Onnela JP, Saramäki J, Kertész J, Kaski K. Intensity and coherence of motifs in weighted complex networks. Phys Rev E Stat Nonlin Soft Matter Phys. 2005;71(6, pt 2):065103. doi:10.1103/PhysRevE.71.065103 16089800

[39] Humphries MD, Gurney K. Network “small-world-ness”: a quantitative method for determining canonical network equivalence. PLoS One. 2008;3(4):e0002051. doi:10.1371/journal.pone.0002051 18446219 PMC2323569

[40] Achard S, Bullmore E. Efficiency and cost of economical brain functional networks. PLoS Comput Biol. 2007;3(2):e17. doi:10.1371/journal.pcbi.0030017 17274684 PMC1794324

[41] Winkler AM, Ridgway GR, Webster MA, Smith SM, Nichols TE. Permutation inference for the general linear model. Neuroimage. 2014;92(100):381-397. doi:10.1016/j.neuroimage.2014.01.060 24530839 PMC4010955

[42] Benjamini Y, Hochberg Y. Controlling the false discovery rate: a practical and powerful approach to multiple testing. J R Stat Soc B. 1995;57(1):289-300. doi:10.1111/j.2517-6161.1995.tb02031.x

[43] Freedman D, Lane D. A nonstochastic interpretation of reported significance levels. J Bus Econ Stat. 1983;1(4):292-298. doi:10.1080/07350015.1983.10509354

[44] Mouka V, Drougia A, Xydis VG, . Functional and structural connectivity of the brain in very preterm babies: relationship with gestational age and body and brain growth. Pediatr Radiol. 2019;49(8):1078-1084. doi:10.1007/s00247-019-04412-6 31053875

[45] Smyser CD, Snyder AZ, Shimony JS, Mitra A, Inder TE, Neil JJ. Resting-state network complexity and magnitude are reduced in prematurely born infants. Cereb Cortex. 2016;26(1):322-333. doi:10.1093/cercor/bhu251 25331596 PMC4677980

[46] Wehrle FM, Michels L, Guggenberger R, . Altered resting-state functional connectivity in children and adolescents born very preterm short title. Neuroimage Clin. 2018;20:1148-1156. doi:10.1016/j.nicl.2018.10.002 30388598 PMC6214877

[47] Scheinost D, Kwon SH, Shen X, . Preterm birth alters neonatal, functional rich club organization. Brain Struct Funct. 2016;221(6):3211-3222. doi:10.1007/s00429-015-1096-6 26341628 PMC4779074

[48] Liu J, Qin W, Zhang J, Zhang X, Yu C. Enhanced interhemispheric functional connectivity compensates for anatomical connection damages in subcortical stroke. Stroke. 2015;46(4):1045-1051. doi:10.1161/STROKEAHA.114.007044 25721013

[49] Macdonald RL, Curry DJ, Aihara Y, Zhang ZD, Jahromi BS, Yassari R. Magnesium and experimental vasospasm. J Neurosurg. 2004;100(1):106-110. doi:10.3171/jns.2004.100.1.0106 14743919

[50] Rantone TH, Grönlund JU, Jalonen JO, . Comparison of the effects of antenatal magnesium sulphate and ritodrine exposure on circulatory adaptation in preterm infants. Clin Physiol Funct Imaging. 2002;22(1):13-17. doi:10.1046/j.1475-097X.2002.00387.x 12003092

[51] Maknojia S, Churchill NW, Schweizer TA, Graham SJ. Resting state fMRI: going through the motions. Front Neurosci. 2019;13:825. doi:10.3389/fnins.2019.00825 31456656 PMC6700228

[52] van den Heuvel MP, Kersbergen KJ, de Reus MA, . The neonatal connectome during preterm brain development. Cereb Cortex. 2015;25(9):3000-3013. doi:10.1093/cercor/bhu095 24833018 PMC4537441

[53] De Asis-Cruz J, Kapse K, Basu SK, . Functional brain connectivity in ex utero premature infants compared to in utero fetuses. Neuroimage. 2020;219:117043. doi:10.1016/j.neuroimage.2020.117043 32534962 PMC7493786

