# Supplementary material for: Prenatal Magnesium Sulfate and Functional Connectivity in Offspring at Term-Equivalent Age
Source: JAMA Netw Open. 2024 May 28;7(5):e2413508. doi: 10.1001/jamanetworkopen.2024.13508 (PMC11134217; doi:10.1001/jamanetworkopen.2024.13508)
Supplement: Supplement 1. — eTable 1. Characteristics of Mothers and Infants Included in and Excluded From the Resting-State fMRI Analyses eTable 2. Volumes of Voxels in Which a Significant (P < .05) Association Between Voxel Mean Connectivity and Magnesium Sulfate (MgSO4) Was Detected eFigure 1. Region Mean Connectivity and Connectivity Between Regions eTable 3. Comparison of Global Network Metrics Between Treatment Groups in Alternate Set of Networks eTable 4. Comparison of Global Network Metrics Between Treatment Groups Within the Largest MRI Site (n = 32) eFigure 2. Network Metrics at Each Density Threshold eTable 5. Functional Connectivity at Each Node eTable 6. Clustering Coefficient AUC at Each Node eTable 7. Local Efficiency AUC at Each Node [file jamanetwopen-e2413508-s001.pdf]

Supplementary Online Content

Ufkes S, Kennedy E, Poppe T, et al. Prenatal magnesium and functional connectivity in offspring at term-equivalent age. *JAMA Netw Open*. 2024;7(5):e2413508. doi:10.1001/jamanetworkopen.2024.13508

eTable 1. Characteristics of Mothers and Infants Included in and Excluded From the Resting-State fMRI Analyses
eTable 2. Volumes of Voxels in Which a Significant (*P* < .05) Association Between Voxel Mean Connectivity and Magnesium Sulfate ($MgSO_4$) Was Detected
eFigure 1. Region Mean Connectivity and Connectivity Between Regions
eTable 3. Comparison of Global Network Metrics Between Treatment Groups in Alternate Set of Networks
eTable 4. Comparison of Global Network Metrics Between Treatment Groups Within the Largest MRI Site (n = 32)
eFigure 2. Network Metrics at Each Density Threshold
eTable 5. Functional Connectivity at Each Node
eTable 6. Clustering Coefficient AUC at Each Node
eTable 7. Local Efficiency AUC at Each Node

This supplementary material has been provided by the authors to give readers additional information about their work.

© 2024 Ufkes S et al. *JAMA Network Open*.

eTable 1. Characteristics of Mothers and Infants Included in and Excluded From the Resting-State fMRI Analyses

Characteristics	Included in analyses	Not included in analyses	P value
Mothers	n=40	n=98	N/A
Age (years): Mean (SD)	30.4 (6.3)	31.9 (6.2)	0.20
Parity: N (%)	21 (52.5)	60 (61.2)	0.35
Ethnicity: N (%)			
. Caucasian	14 (35)	49 (50)	0.57
. Asian	9 (22.5)	18 (18.4)	
. Polynesian	4 (10)	5 (5.1)	
. Maori	5 (12.5)	10 (10.2)	
. Other	8 (20)	16 (16.3)	
BMI (kg/m^2): Mean (SD)	28 (7)	26.7 (6.4)	0.33
GA at entry (weeks): Median (IQ range)	32 (31.1 - 32.95)	32 (31.0 - 32.7)	0.74
Main risk for preterm birth:			
. Antepartum Haemorrhage: N (%)	4 (10)	13 (13.3)	0.78
. PPROM: N (%)	11 (27.5)	36 (36.7)	
. Preterm labour: N (%)	18 (45)	41 (41.8)	
. Pre-eclampsia: N (%)	8 (20)	17 (17.4)	
. Fetal compromise: N (%)	4 (10)	22 (22.5)	
. Other: N (%)	7 (17.5)	20 (20.4)	
Received Allocated Treatment: N (%)	40 (100)	94 (95.9)	0.25
Infants	n=45	n=114	N/A
Received $MgSO_4$: N (%)	24 (53.3)	49 (43.0)	0.24
GA at birth (weeks): Median (IQ range)	32 (31.3 - 33.0)	32 (31.1 - 32.6)	0.60
Twins: N (%)			
. Singleton	33 (73.3)	83 (72.8)	0.85
. Twin 1	7 (15.6)	15 (13.2)	
. Twin 2	5 (11.1)	16 (14)	
MRI site: N (%)			
. Auckland/CAMRI	32 (71.1)	71 (62.3)	0.63
. Christchurch MRI	10 (22.2)	31 (27.2)	
. Adelaide/SAHMRI	3 (6.7)	12 (10.5)	
PMA at MRI (weeks): Median (IQ range)	40 (39.1 - 41.1)	40 (39.2 - 41.3)	0.51
Birth weight (g): Mean (SD)	1817 (510)	1732 (444)	0.30
Birth weight (z score): Mean (SD)	0.2 (1.1)	0 (1.1)	0.29
Bronchopulmonary dysplasia: N (%)	4 (8.9)	9 (7.9)	1.00
Necrotising enterocolitis: N (%)	0 (0)	0 (0)	N/A
Full breastmilk feeding at discharge: N (%)	25 (71.4)	86 (86)	0.07
Sex (female): N (%)	23 (51.1)	49 (43)	0.38

$MgSO_4$ = magnesium sulphate; BMI = body mass index; GA = gestational age; PPROM = preterm prelabour rupture of the membranes; PMA = postmenstrual age

© 2024 Ufkes S et al. *JAMA Network Open*.

eTable 2. Volumes of Voxels in Which a Significant (*P* < .05) Association Between Voxel Mean Connectivity and Magnesium Sulfate ($MgSO_4$) Was Detected

Region	Hemisphere	Abbreviation	Volume of region (mm^3)	Volume of significant voxels (mm^3)
Precentral gyrus	left	PreCG-L	8336	0
Precentral gyrus	right	PreCG-R	7120	0
Superior frontal gyrus (dorsal)	left	SFGdor-L	8896	0
Superior frontal gyrus (dorsal)	right	SFGdor-R	7568	0
Orbitofrontal cortex (superior)	left	ORBsupb-L	2488	0
Orbitofrontal cortex (superior)	right	ORBsupb-R	2984	0
Middle frontal gyrus	left	MFG-L	16576	0
Middle frontal gyrus	right	MFG-R	16592	0
Orbitofrontal cortex (middle)	left	ORBmid-L	2528	0
Orbitofrontal cortex (middle)	right	ORBmid-R	3360	0
Inferior frontal gyrus (opercular)	left	IFGoperc-L	2432	0
Inferior frontal gyrus (opercular)	right	IFGoperc-R	3432	88
Inferior frontal gyrus (triangular)	left	IFGtriang-L	6824	0
Inferior frontal gyrus (triangular)	right	IFGtriang-R	5888	32
Orbitofrontal cortex (inferior)	left	ORBinf-L	5544	0
Orbitofrontal cortex (inferior)	right	ORBinf-R	5448	96
Rolandic operculum	left	ROL-L	2976	0
Rolandic operculum	right	ROL-R	3744	32
Supplementary motor area	left	SMA-L	4160	0
Supplementary motor area	right	SMA-R	3784	0
Olfactory	left	OLF-L	1112	0
Olfactory	right	OLF-R	1152	0
Superior frontal gyrus (medial)	left	SFGmed-L	8072	0
Superior frontal gyrus (medial)	right	SFGmed-R	5664	0
Orbitofrontal cortex (medial)	left	ORBmed-L	1944	0
Orbitofrontal cortex (medial)	right	ORBmed-R	2816	0
Rectus gyrus	left	REC-L	2368	0
Rectus gyrus	right	REC-R	2040	0
Insula	left	INS-L	4688	0
Insula	right	INS-R	4312	176
Anterior cingulate gyrus	left	ACG-L	4288	0
Anterior cingulate gyrus	right	ACG-R	4216	0
Middle cingulate gyrus	left	MCG-L	5712	0
Middle cingulate gyrus	right	MCG-R	6264	0
Posterior cingulate gyrus	left	PCG-L	1096	0
Posterior cingulate gyrus	right	PCG-R	656	0
Hippocampus	left	HIP-L	2488	0
Hippocampus	right	HIP-R	2296	8
ParaHippocampal gyrus	left	PHG-L	3336	0
ParaHippocampal gyrus	right	PHG-R	3632	192
Amygdala	left	AMYG-L	632	0
Amygdala	right	AMYG-R	664	344
Calcarine cortex	left	CAL-L	8000	120
Calcarine cortex	right	CAL-R	5696	336
Cuneus	left	CUN-L	5632	568
Cuneus	right	CUN-R	5176	384
Lingual gyrus	left	LING-L	6888	128
Lingual gyrus	right	LING-R	6912	1344

© 2024 Ufkes S et al. *JAMA Network Open*.

**Region**	**Hemisphere**	**Abbreviation**	**Volume of region (mm^3)**	**Volume of significant voxels (mm^3)**
Superior occipital gyrus	left	SOG-L	4696	592
Superior occipital gyrus	right	SOG-R	3600	408
Middle occipital gyrus	left	MOG-L	9832	360
Middle occipital gyrus	right	MOG-R	6928	1264
Inferior occipital gyrus	left	IOG-L	2600	0
Inferior occipital gyrus	right	IOG-R	2424	160
Fusiform gyrus	left	FFG-L	8512	0
Fusiform gyrus	right	FFG-R	8768	968
Postcentral gyrus	left	PoCG-L	9592	0
Postcentral gyrus	right	PoCG-R	8216	0
Superior parietal gyrus	left	SPG-L	5040	0
Superior parietal gyrus	right	SPG-R	5824	56
Inferior parietal lobule	left	IPL-L	8320	0
Inferior parietal lobule	right	IPL-R	5440	192
Supramarginal gyrus	left	SMG-L	4160	0
Supramarginal gyrus	right	SMG-R	6272	640
Angular gyrus	left	ANG-L	4840	0
Angular gyrus	right	ANG-R	7392	200
Precuneus	left	PCUN-L	11792	104
Precuneus	right	PCUN-R	8472	64
Paracentral lobule	left	PCL-L	1544	0
Paracentral lobule	right	PCL-R	776	0
Caudate	left	CAU-L	2296	0
Caudate	right	CAU-R	2488	0
Putamen	left	PUT-L	3432	0
Putamen	right	PUT-R	3432	120
Pallidum	left	PAL-L	872	0
Pallidum	right	PAL-R	944	280
Thalamus	left	THA-L	3168	0
Thalamus	right	THA-R	3048	8
Heschl gyrus	left	HES-L	528	0
Heschl gyrus	right	HES-R	456	0
Superior temporal gyrus	left	STG-L	9024	0
Superior temporal gyrus	right	STG-R	9128	1776
Temporal pole (superior)	left	TPOsup-L	4816	0
Temporal pole (superior)	right	TPOsup-R	5032	1576
Middle temporal gyrus	left	MTG-L	15992	0
Middle temporal gyrus	right	MTG-R	16040	2976
Temporal pole (middle)	left	TPOmid-L	3144	0
Temporal pole (middle)	right	TPOmid-R	3536	216
Inferior temporal gyrus	left	ITG-L	8880	0
Inferior temporal gyrus	right	ITG-R	11232	536
Cerebellar hemisphere	left	CBH-L	11216	8
Cerebellar hemisphere	right	CBH-R	10104	392

© 2024 Ufkes S et al. *JAMA Network Open*.

eFigure 1. Region Mean Connectivity and Connectivity Between Regions

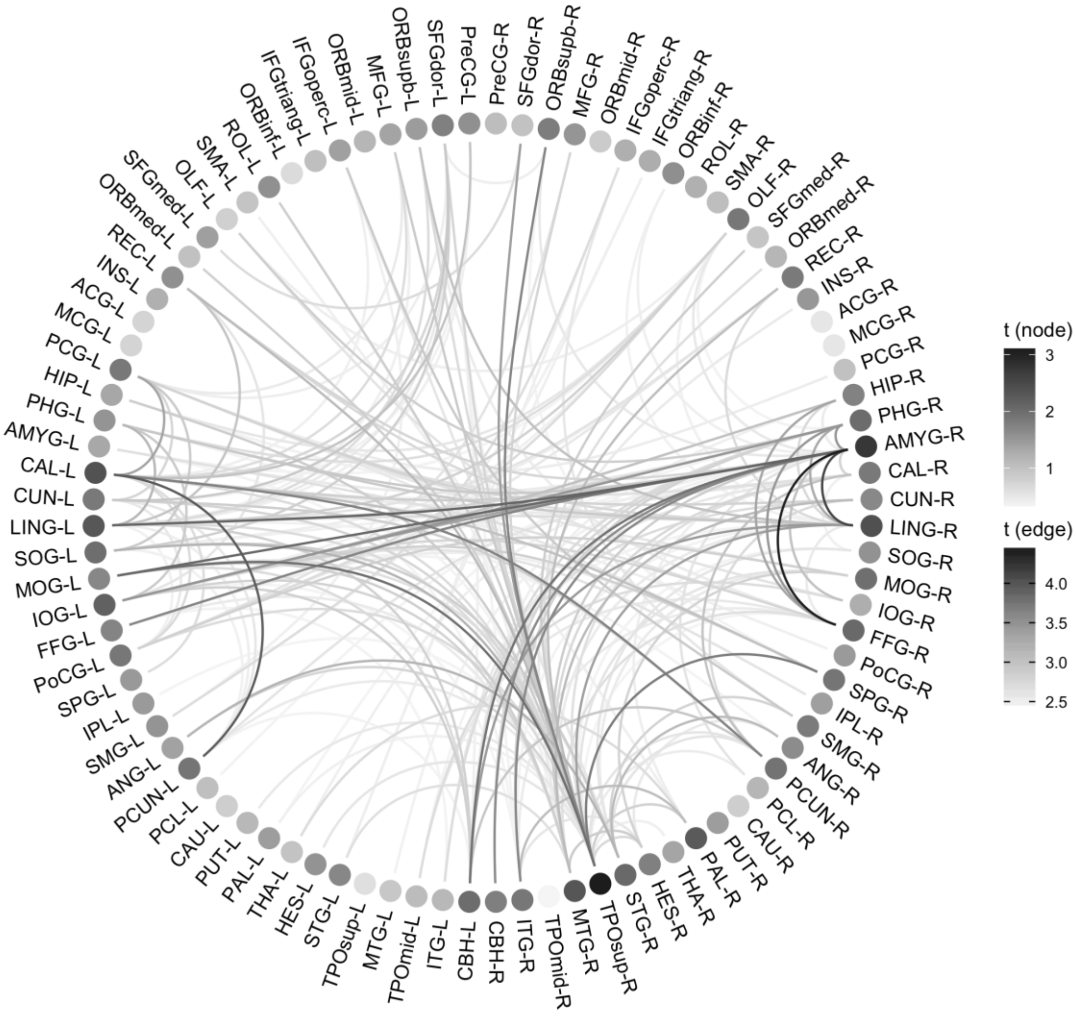

Magnesium sulphate ($MgSO_4$) is associated with increased connection strength between many grey matter regions. Each circle represents an anatomical region. The darkness of each circle represents the *t* value comparing the region mean connectivity between treatment groups. The lines indicate the pairs of regions between which connectivity is most strongly associated with $MgSO_4$. The darkness of each line represents the *t* value for the group comparison of connectivity between the two regions. For visual clarity, lines are only drawn for the highest 5% of connection *t* values. PreCG-L/R=Precentral gyrus; SFGdor-L/R=Superior frontal gyrus (dorsal); ORBsupb-L/R=Orbitofrontal cortex (superior); MFG-L/R=Middle frontal gyrus; ORBmid-L/R=Orbitofrontal cortex (middle); IFGoperc-L/R=Inferior frontal gyrus (opercular); IFGtriang-L/R=Inferior frontal gyrus (triangular); ORBinf-L/R=Orbitofrontal cortex (inferior); ROL-L/R=Rolandic operculum; SMA-L/R=Supplementary motor area; OLF-L/R=Olfactory; SFGmed-L/R=Superior frontal gyrus (medial); ORBmed-L/R=Orbitofrontal cortex (medial); REC-L/R=Rectus gyrus; INS-L/R=Insula; ACG-L/R=Anterior cingulate gyrus; MCG-L/R=Middle cingulate gyrus; PCG-L/R=Posterior cingulate gyrus; HIP-L/R=Hippocampus; PHG-L/R=ParaHippocampal gyrus; AMYG-L/R=Amygdala; CAL-L/R=Calcarine cortex; CUN-L/R=Cuneus; LING-L/R=Lingual gyrus; SOG-L/R=Superior occipital gyrus; MOG-L/R=Middle occipital gyrus; IOG-L/R=Inferior occipital gyrus; FFG-L/R=Fusiform gyrus; PoCG-L/R=Postcentral gyrus; SPG-L/R=Superior parietal gyrus; IPL-L/R=Inferior parietal lobule; SMG-

© 2024 Ufkes S et al. *JAMA Network Open*.

L/R=Supramarginal gyrus; ANG-L/R=Angular gyrus; PCUN-L/R=Precuneus; PCL-L/R=Paracentral lobule; CAU-L/R=Caudate; PUT-L/R=Putamen; PAL-L/R=Pallidum; THA-L/R=Thalamus; HES-L/R=Heschl gyrus; STG-L/R=Superior temporal gyrus; TPOsup-L/R=Temporal pole (superior); MTG-L/R=Middle temporal gyrus; TPOmid-L/R=Temporal pole (middle); ITG-L/R=Inferior temporal gyrus; CBH-L/R=Cerebellar hemisphere

© 2024 Ufkes S et al. *JAMA Network Open*.

eTable 3. Comparison of Global Network Metrics Between Treatment Groups in Alternate Set of Networks

**Metric**	**Placebo**	**$MgSO_4$**	***g***	***p***
Characteristic path length	2.50 ± 0.56	2.31 ± 0.56	-0.35 (-0.94 – 0.25)	**0.04**
Global efficiency	0.43 ± 0.11	0.47 ± 0.13	0.32 (-0.28 – 0.91)	0.05
Clustering coefficient	0.49 ± 0.17	0.57 ± 0.20	0.42 (-0.18 – 1.02)	**0.02**
Transitivity	0.50 ± 0.19	0.59 ± 0.22	0.45 (-0.15 – 1.05)	**0.02**
Local efficiency	0.68 ± 0.21	0.77 ± 0.23	0.37 (-0.22 – 0.97)	**0.03**
Modularity	0.30 ± 0.05	0.31 ± 0.07	0.14 (-0.46 – 0.73)	0.75
Small worldness	1.40 ± 0.24	1.41 ± 0.28	0.05 (-0.54 – 0.64)	0.94

In the alternate set of networks, edge weights were defined as the positive part of Fisher z-transformed Pearson correlation coefficients. Group values represent the mean ± standard deviation of the metric AUC. Effect size is reported as Hedge's *g* (95% confidence interval). P values less than 0.05 are bolded.

© 2024 Ufkes S et al. *JAMA Network Open*.

eTable 4. Comparison of Global Network Metrics Between Treatment Groups Within the Largest MRI Site (n = 32)

**Metric**	**Placebo (n=14)**	**$MgSO_4$ (n=18)**	***g***	***p***
Characteristic path length	3.18 ± 0.41	3.02 ± 0.39	-0.39 (-1.11 – 0.32)	**0.04**
Global efficiency	0.32 ± 0.05	0.34 ± 0.05	0.39 (-0.33 – 1.11)	**0.04**
Clustering coefficient	0.35 ± 0.0c8	0.40 ± 0.09	0.54 (-0.19 – 1.26)	**0.02**
Transitivity	0.36 ± 0.09	0.42 ± 0.11	0.57 (-0.15 – 1.30)	**0.01**
Local efficiency	0.50 ± 0.09	0.55 ± 0.09	0.47 (-0.25 – 1.19)	**0.03**
Modularity	0.29 ± 0.05	0.30 ± 0.07	0.10 (-0.61 – 0.81)	0.63
Small worldness	1.43 ± 0.24	1.44 ± 0.30	0.05 (-0.66 – 0.76)	0.55

Group values represent the mean ± standard deviation of the metric AUC. Effect size is reported as Hedge's *g* (95% confidence interval). P values less than 0.05 are bolded.

© 2024 Ufkes S et al. *JAMA Network Open*.

eFigure 2. Network Metrics at Each Density Threshold

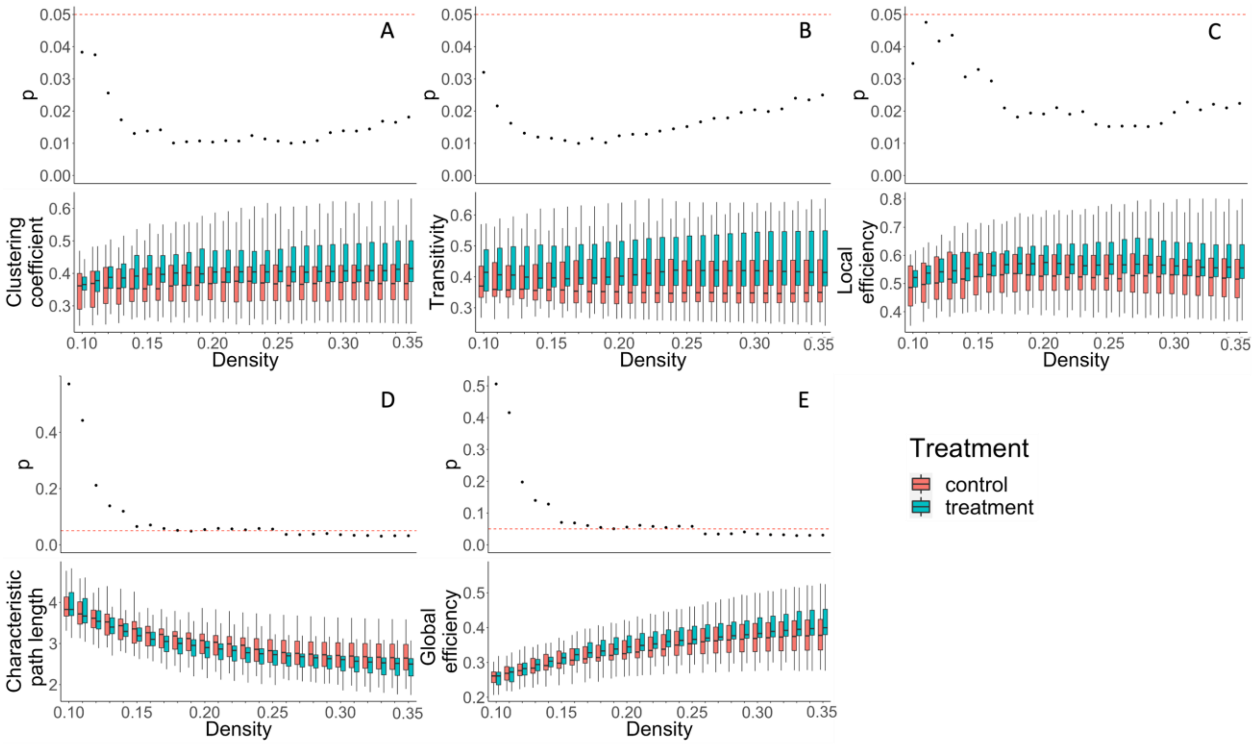

Comparison of (A) clustering coefficient; (B) transitivity; (C) local efficiency; (D) characteristic path length; and (E) global efficiency at each network density threshold. The top panel in each subplot shows the *p* value for the test comparing the $MgSO_4$ and placebo groups, with a red dashed line at $p = 0.05$.

© 2024 Ufkes S et al. *JAMA Network Open*.

eTable 5. Functional Connectivity at Each Node

Region	Hemisphere	Abbreviation	Placebo	$MgSO_4$	*g*	*p*
Precentral gyrus	left	PreCG-L	0.43 ± 0.16	0.48 ± 0.17	0.26 (-0.33 – 0.86)	0.28
Precentral gyrus	right	PreCG-R	0.45 ± 0.16	0.46 ± 0.19	0.07 (-0.52 – 0.66)	0.38
Superior frontal gyrus (dorsal)	left	SFGdor-L	0.42 ± 0.16	0.48 ± 0.16	0.36 (-0.24 – 0.95)	0.27
Superior frontal gyrus (dorsal)	right	SFGdor-R	0.44 ± 0.16	0.46 ± 0.17	0.14 (-0.45 – 0.73)	0.39
Orbitofrontal cortex (superior)	left	ORBsupb-L	0.39 ± 0.18	0.43 ± 0.16	0.25 (-0.34 – 0.85)	0.28
Orbitofrontal cortex (superior)	right	ORBsupb-R	0.38 ± 0.17	0.44 ± 0.16	0.35 (-0.25 – 0.95)	0.27
Middle frontal gyrus	left	MFG-L	0.40 ± 0.15	0.45 ± 0.17	0.30 (-0.29 – 0.90)	0.29
Middle frontal gyrus	right	MFG-R	0.42 ± 0.17	0.46 ± 0.16	0.25 (-0.34 – 0.85)	0.28
Orbitofrontal cortex (middle)	left	ORBmid-L	0.36 ± 0.17	0.38 ± 0.17	0.12 (-0.47 – 0.71)	0.36
Orbitofrontal cortex (middle)	right	ORBmid-R	0.37 ± 0.14	0.40 ± 0.18	0.14 (-0.45 – 0.74)	0.45
Inferior frontal gyrus (opercular)	left	IFGoperc-L	0.41 ± 0.17	0.45 ± 0.17	0.22 (-0.37 – 0.82)	0.28
Inferior frontal gyrus (opercular)	right	IFGoperc-R	0.43 ± 0.15	0.45 ± 0.18	0.11 (-0.48 – 0.70)	0.32
Inferior frontal gyrus (triangular)	left	IFGtriang-L	0.40 ± 0.17	0.42 ± 0.18	0.13 (-0.46 – 0.72)	0.39
Inferior frontal gyrus (triangular)	right	IFGtriang-R	0.40 ± 0.14	0.43 ± 0.17	0.14 (-0.45 – 0.74)	0.32
Orbitofrontal cortex (inferior)	left	ORBinf-L	0.42 ± 0.17	0.41 ± 0.17	-0.04 (-0.63 – 0.55)	0.55
Orbitofrontal cortex (inferior)	right	ORBinf-R	0.41 ± 0.15	0.44 ± 0.16	0.23 (-0.36 – 0.83)	0.28
Rolandic operculum	left	ROL-L	0.42 ± 0.15	0.46 ± 0.17	0.22 (-0.38 – 0.81)	0.28
Rolandic operculum	right	ROL-R	0.41 ± 0.16	0.43 ± 0.19	0.11 (-0.48 – 0.70)	0.32
Supplementary motor area	left	SMA-L	0.40 ± 0.17	0.41 ± 0.18	0.10 (-0.49 – 0.69)	0.40
Supplementary motor area	right	SMA-R	0.41 ± 0.16	0.42 ± 0.19	0.06 (-0.53 – 0.65)	0.39
Olfactory	left	OLF-L	0.37 ± 0.18	0.39 ± 0.18	0.10 (-0.49 – 0.69)	0.47
Olfactory	right	OLF-R	0.34 ± 0.17	0.41 ± 0.18	0.37 (-0.22 – 0.97)	0.27
Superior frontal gyrus (medial)	left	SFGmed-L	0.41 ± 0.15	0.45 ± 0.16	0.24 (-0.35 – 0.84)	0.28
Superior frontal gyrus (medial)	right	SFGmed-R	0.41 ± 0.16	0.44 ± 0.16	0.18 (-0.42 – 0.77)	0.40
Orbitofrontal cortex (medial)	left	ORBmed-L	0.41 ± 0.16	0.43 ± 0.18	0.11 (-0.49 – 0.70)	0.39
Orbitofrontal cortex (medial)	right	ORBmed-R	0.42 ± 0.15	0.45 ± 0.16	0.19 (-0.40 – 0.79)	0.36
Rectus gyrus	left	REC-L	0.38 ± 0.17	0.43 ± 0.16	0.29 (-0.31 – 0.88)	0.28
Rectus gyrus	right	REC-R	0.36 ± 0.18	0.42 ± 0.17	0.36 (-0.23 – 0.96)	0.27
Insula	left	INS-L	0.44 ± 0.16	0.47 ± 0.17	0.13 (-0.46 – 0.73)	0.32
Insula	right	INS-R	0.42 ± 0.17	0.46 ± 0.19	0.24 (-0.35 – 0.83)	0.28
Anterior cingulate gyrus	left	ACG-L	0.45 ± 0.16	0.45 ± 0.17	0.04 (-0.55 – 0.64)	0.49
Anterior cingulate gyrus	right	ACG-R	0.43 ± 0.17	0.43 ± 0.17	0.00 (-0.59 – 0.59)	0.61
Middle cingulate gyrus	left	MCG-L	0.48 ± 0.15	0.48 ± 0.18	0.03 (-0.57 – 0.62)	0.48
Middle cingulate gyrus	right	MCG-R	0.48 ± 0.15	0.48 ± 0.20	-0.03 (-0.62 – 0.56)	0.61
Posterior cingulate gyrus	left	PCG-L	0.40 ± 0.14	0.44 ± 0.17	0.27 (-0.33 – 0.86)	0.27
Posterior cingulate gyrus	right	PCG-R	0.41 ± 0.17	0.42 ± 0.20	0.04 (-0.55 – 0.63)	0.39
Hippocampus	left	HIP-L	0.39 ± 0.18	0.43 ± 0.18	0.17 (-0.42 – 0.77)	0.30
Hippocampus	right	HIP-R	0.40 ± 0.17	0.44 ± 0.19	0.25 (-0.34 – 0.85)	0.27
ParaHippocampal gyrus	left	PHG-L	0.37 ± 0.17	0.42 ± 0.17	0.27 (-0.33 – 0.86)	0.28
ParaHippocampal gyrus	right	PHG-R	0.38 ± 0.17	0.43 ± 0.18	0.31 (-0.29 – 0.90)	0.27
Amygdala	left	AMYG-L	0.32 ± 0.19	0.37 ± 0.19	0.25 (-0.34 – 0.85)	0.30
Amygdala	right	AMYG-R	0.31 ± 0.15	0.40 ± 0.17	0.58 (-0.02 – 1.19)	0.27
Calcarine cortex	left	CAL-L	0.38 ± 0.19	0.45 ± 0.17	0.39 (-0.21 – 0.98)	0.27
Calcarine cortex	right	CAL-R	0.40 ± 0.16	0.44 ± 0.18	0.22 (-0.38 – 0.81)	0.27
Cuneus	left	CUN-L	0.41 ± 0.16	0.45 ± 0.17	0.28 (-0.32 – 0.87)	0.27
Cuneus	right	CUN-R	0.42 ± 0.15	0.45 ± 0.17	0.21 (-0.39 – 0.80)	0.27
Lingual gyrus	left	LING-L	0.37 ± 0.18	0.45 ± 0.18	0.43 (-0.17 – 1.03)	0.27
Lingual gyrus	right	LING-R	0.38 ± 0.15	0.44 ± 0.18	0.40 (-0.20 – 1.00)	0.27
Superior occipital gyrus	left	SOG-L	0.41 ± 0.15	0.47 ± 0.15	0.38 (-0.21 – 0.98)	0.27
Superior occipital gyrus	right	SOG-R	0.40 ± 0.15	0.45 ± 0.17	0.27 (-0.32 – 0.87)	0.28
Middle occipital gyrus	left	MOG-L	0.42 ± 0.16	0.45 ± 0.16	0.24 (-0.36 – 0.83)	0.27
Middle occipital gyrus	right	MOG-R	0.35 ± 0.12	0.41 ± 0.16	0.38 (-0.21 – 0.98)	0.27
Inferior occipital gyrus	left	IOG-L	0.28 ± 0.15	0.34 ± 0.16	0.35 (-0.25 – 0.94)	0.27
Inferior occipital gyrus	right	IOG-R	0.24 ± 0.09	0.27 ± 0.14	0.26 (-0.33 – 0.86)	0.32
Fusiform gyrus	left	FFG-L	0.37 ± 0.17	0.41 ± 0.18	0.21 (-0.39 – 0.80)	0.27
Fusiform gyrus	right	FFG-R	0.37 ± 0.16	0.41 ± 0.16	0.24 (-0.36 – 0.83)	0.27
Postcentral gyrus	left	PoCG-L	0.41 ± 0.16	0.46 ± 0.17	0.34 (-0.26 – 0.94)	0.27
Postcentral gyrus	right	PoCG-R	0.41 ± 0.16	0.44 ± 0.19	0.17 (-0.42 – 0.76)	0.28
Superior parietal gyrus	left	SPG-L	0.41 ± 0.17	0.45 ± 0.17	0.28 (-0.32 – 0.87)	0.28
Superior parietal gyrus	right	SPG-R	0.38 ± 0.16	0.45 ± 0.15	0.44 (-0.16 – 1.04)	0.27
Inferior parietal lobule	left	IPL-L	0.43 ± 0.15	0.46 ± 0.17	0.22 (-0.37 – 0.82)	0.28
Inferior parietal lobule	right	IPL-R	0.41 ± 0.16	0.44 ± 0.17	0.20 (-0.39 – 0.80)	0.28
Supramarginal gyrus	left	SMG-L	0.39 ± 0.17	0.44 ± 0.18	0.27 (-0.33 – 0.86)	0.28

© 2024 Ufkes S et al. *JAMA Network Open*.

Region	Hemisphere	Abbreviation	Placebo	$MgSO_4$	*g*	*p*
Supramarginal gyrus	right	SMG-R	0.38 ± 0.16	0.43 ± 0.18	0.29 (-0.31 – 0.88)	0.27
Angular gyrus	left	ANG-L	0.41 ± 0.16	0.44 ± 0.16	0.20 (-0.39 – 0.80)	0.28
Angular gyrus	right	ANG-R	0.41 ± 0.15	0.45 ± 0.16	0.25 (-0.34 – 0.85)	0.28
Precuneus	left	PCUN-L	0.47 ± 0.16	0.51 ± 0.15	0.25 (-0.34 – 0.85)	0.27
Precuneus	right	PCUN-R	0.46 ± 0.17	0.50 ± 0.17	0.23 (-0.36 – 0.83)	0.27
Paracentral lobule	left	PCL-L	0.36 ± 0.17	0.37 ± 0.18	0.05 (-0.54 – 0.64)	0.39
Paracentral lobule	right	PCL-R	0.38 ± 0.16	0.39 ± 0.18	0.06 (-0.53 – 0.66)	0.36
Caudate	left	CAU-L	0.42 ± 0.16	0.44 ± 0.19	0.09 (-0.50 – 0.68)	0.45
Caudate	right	CAU-R	0.44 ± 0.17	0.45 ± 0.18	0.08 (-0.51 – 0.67)	0.45
Putamen	left	PUT-L	0.43 ± 0.16	0.46 ± 0.19	0.14 (-0.45 – 0.74)	0.36
Putamen	right	PUT-R	0.42 ± 0.16	0.46 ± 0.19	0.23 (-0.37 – 0.82)	0.28
Pallidum	left	PAL-L	0.37 ± 0.18	0.42 ± 0.19	0.23 (-0.36 – 0.82)	0.28
Pallidum	right	PAL-R	0.35 ± 0.17	0.43 ± 0.18	0.42 (-0.18 – 1.01)	0.27
Thalamus	left	THA-L	0.43 ± 0.17	0.45 ± 0.20	0.10 (-0.49 – 0.69)	0.39
Thalamus	right	THA-R	0.41 ± 0.17	0.45 ± 0.19	0.18 (-0.42 – 0.77)	0.29
Heschl gyrus	left	HES-L	0.36 ± 0.17	0.41 ± 0.19	0.22 (-0.37 – 0.82)	0.28
Heschl gyrus	right	HES-R	0.32 ± 0.17	0.37 ± 0.18	0.28 (-0.32 – 0.87)	0.27
Superior temporal gyrus	left	STG-L	0.40 ± 0.16	0.44 ± 0.18	0.24 (-0.35 – 0.84)	0.27
Superior temporal gyrus	right	STG-R	0.39 ± 0.16	0.45 ± 0.18	0.32 (-0.27 – 0.92)	0.27
Temporal pole (superior)	left	TPOsup-L	0.37 ± 0.15	0.36 ± 0.16	-0.01 (-0.61 – 0.58)	0.56
Temporal pole (superior)	right	TPOsup-R	0.32 ± 0.12	0.41 ± 0.15	0.62 (0.01 – 1.22)	0.27
Middle temporal gyrus	left	MTG-L	0.43 ± 0.16	0.44 ± 0.19	0.03 (-0.57 – 0.62)	0.41
Middle temporal gyrus	right	MTG-R	0.39 ± 0.13	0.45 ± 0.18	0.37 (-0.23 – 0.97)	0.27
Temporal pole (middle)	left	TPOmid-L	0.30 ± 0.14	0.33 ± 0.17	0.19 (-0.40 – 0.79)	0.38
Temporal pole (middle)	right	TPOmid-R	0.31 ± 0.16	0.30 ± 0.16	-0.04 (-0.63 – 0.55)	0.74
Inferior temporal gyrus	left	ITG-L	0.31 ± 0.15	0.33 ± 0.18	0.10 (-0.50 – 0.69)	0.36
Inferior temporal gyrus	right	ITG-R	0.35 ± 0.12	0.38 ± 0.15	0.24 (-0.36 – 0.83)	0.27
Cerebellar hemisphere	left	CBH-L	0.34 ± 0.15	0.39 ± 0.18	0.28 (-0.32 – 0.87)	0.27
Cerebellar hemisphere	right	CBH-R	0.34 ± 0.15	0.38 ± 0.18	0.25 (-0.35 – 0.84)	0.27

Treatment group values represent the mean ± standard deviation. Effect size is reported as Hedge's *g* (95% confidence interval). The *p* value is for the test that the functional connectivity at the node differs between treatment groups, adjusted for multiple comparisons across all nodes.

© 2024 Ufkes S et al. *JAMA Network Open*.

eTable 6. Clustering Coefficient AUC at Each Node

Region	Hemisphere	Abbreviation	Placebo	$MgSO_4$	*g*	*p*
Precentral gyrus	left	PreCG-L	0.39 ± 0.10	0.42 ± 0.11	0.29 (-0.30 – 0.89)	0.15
Precentral gyrus	right	PreCG-R	0.36 ± 0.13	0.41 ± 0.14	0.39 (-0.20 – 0.99)	0.22
Superior frontal gyrus (dorsal)	left	SFGdor-L	0.43 ± 0.13	0.43 ± 0.13	0.04 (-0.55 – 0.63)	0.46
Superior frontal gyrus (dorsal)	right	SFGdor-R	0.40 ± 0.12	0.44 ± 0.10	0.36 (-0.24 – 0.96)	0.28
Orbitofrontal cortex (superior)	left	ORBsupb-L	0.38 ± 0.14	0.44 ± 0.15	0.41 (-0.18 – 1.01)	0.26
Orbitofrontal cortex (superior)	right	ORBsupb-R	0.43 ± 0.14	0.41 ± 0.17	-0.11 (-0.70 – 0.48)	0.83
Middle frontal gyrus	left	MFG-L	0.39 ± 0.16	0.43 ± 0.10	0.26 (-0.33 – 0.85)	0.28
Middle frontal gyrus	right	MFG-R	0.41 ± 0.11	0.45 ± 0.16	0.32 (-0.28 – 0.92)	0.22
Orbitofrontal cortex (middle)	left	ORBmid-L	0.42 ± 0.18	0.48 ± 0.18	0.30 (-0.30 – 0.90)	0.34
Orbitofrontal cortex (middle)	right	ORBmid-R	0.42 ± 0.17	0.42 ± 0.20	0.01 (-0.58 – 0.60)	0.90
Inferior frontal gyrus (opercular)	left	IFGoperc-L	0.38 ± 0.12	0.45 ± 0.14	0.50 (-0.10 – 1.10)	0.15
Inferior frontal gyrus (opercular)	right	IFGoperc-R	0.40 ± 0.14	0.44 ± 0.16	0.29 (-0.30 – 0.89)	0.28
Inferior frontal gyrus (triangular)	left	IFGtriang-L	0.40 ± 0.15	0.40 ± 0.15	-0.02 (-0.61 – 0.58)	0.95
Inferior frontal gyrus (triangular)	right	IFGtriang-R	0.39 ± 0.14	0.47 ± 0.17	0.48 (-0.12 – 1.08)	0.18
Orbitofrontal cortex (inferior)	left	ORBinf-L	0.33 ± 0.13	0.42 ± 0.14	0.69 (0.08 – 1.30)	0.16
Orbitofrontal cortex (inferior)	right	ORBinf-R	0.37 ± 0.12	0.42 ± 0.16	0.30 (-0.30 – 0.89)	0.30
Rolandic operculum	left	ROL-L	0.35 ± 0.11	0.41 ± 0.14	0.46 (-0.14 – 1.06)	0.21
Rolandic operculum	right	ROL-R	0.37 ± 0.16	0.44 ± 0.17	0.39 (-0.21 – 0.99)	0.22
Supplementary motor area	left	SMA-L	0.45 ± 0.13	0.48 ± 0.19	0.22 (-0.38 – 0.81)	0.26
Supplementary motor area	right	SMA-R	0.43 ± 0.13	0.48 ± 0.16	0.30 (-0.30 – 0.89)	0.29
Olfactory	left	OLF-L	0.38 ± 0.14	0.39 ± 0.20	0.06 (-0.53 – 0.65)	0.90
Olfactory	right	OLF-R	0.41 ± 0.17	0.39 ± 0.20	-0.13 (-0.73 – 0.46)	0.84
Superior frontal gyrus (medial)	left	SFGmed-L	0.46 ± 0.16	0.45 ± 0.12	-0.04 (-0.63 – 0.55)	0.85
Superior frontal gyrus (medial)	right	SFGmed-R	0.45 ± 0.14	0.48 ± 0.13	0.25 (-0.34 – 0.85)	0.30
Orbitofrontal cortex (medial)	left	ORBmed-L	0.39 ± 0.14	0.44 ± 0.18	0.32 (-0.27 – 0.92)	0.23
Orbitofrontal cortex (medial)	right	ORBmed-R	0.37 ± 0.16	0.39 ± 0.16	0.13 (-0.46 – 0.72)	0.69
Rectus gyrus	left	REC-L	0.41 ± 0.16	0.46 ± 0.17	0.31 (-0.29 – 0.90)	0.31
Rectus gyrus	right	REC-R	0.42 ± 0.16	0.47 ± 0.16	0.36 (-0.23 – 0.96)	0.29
Insula	left	INS-L	0.34 ± 0.11	0.38 ± 0.16	0.28 (-0.31 – 0.88)	0.31
Insula	right	INS-R	0.35 ± 0.12	0.42 ± 0.14	0.54 (-0.06 – 1.15)	0.15
Anterior cingulate gyrus	left	ACG-L	0.38 ± 0.11	0.44 ± 0.13	0.50 (-0.10 – 1.10)	0.18
Anterior cingulate gyrus	right	ACG-R	0.39 ± 0.10	0.46 ± 0.14	0.52 (-0.08 – 1.13)	0.15
Middle cingulate gyrus	left	MCG-L	0.35 ± 0.09	0.36 ± 0.13	0.07 (-0.53 – 0.66)	0.31
Middle cingulate gyrus	right	MCG-R	0.35 ± 0.09	0.34 ± 0.14	-0.05 (-0.65 – 0.54)	0.69
Posterior cingulate gyrus	left	PCG-L	0.38 ± 0.15	0.43 ± 0.12	0.37 (-0.23 – 0.97)	0.31
Posterior cingulate gyrus	right	PCG-R	0.40 ± 0.09	0.45 ± 0.20	0.27 (-0.33 – 0.86)	0.31
Hippocampus	left	HIP-L	0.29 ± 0.12	0.38 ± 0.18	0.53 (-0.07 – 1.13)	0.18
Hippocampus	right	HIP-R	0.29 ± 0.12	0.37 ± 0.15	0.51 (-0.09 – 1.11)	0.15
ParaHippocampal gyrus	left	PHG-L	0.32 ± 0.12	0.36 ± 0.20	0.25 (-0.35 – 0.84)	0.37
ParaHippocampal gyrus	right	PHG-R	0.29 ± 0.16	0.36 ± 0.15	0.44 (-0.16 – 1.04)	0.18
Amygdala	left	AMYG-L	0.33 ± 0.22	0.35 ± 0.22	0.07 (-0.53 – 0.66)	0.76
Amygdala	right	AMYG-R	0.30 ± 0.21	0.39 ± 0.22	0.39 (-0.21 – 0.99)	0.21
Calcarine cortex	left	CAL-L	0.43 ± 0.10	0.47 ± 0.13	0.35 (-0.25 – 0.95)	0.30
Calcarine cortex	right	CAL-R	0.40 ± 0.12	0.45 ± 0.13	0.39 (-0.21 – 0.98)	0.28
Cuneus	left	CUN-L	0.43 ± 0.13	0.48 ± 0.11	0.38 (-0.21 – 0.98)	0.28
Cuneus	right	CUN-R	0.40 ± 0.13	0.47 ± 0.11	0.54 (-0.06 – 1.15)	0.15
Lingual gyrus	left	LING-L	0.42 ± 0.11	0.41 ± 0.14	-0.06 (-0.65 – 0.53)	0.77
Lingual gyrus	right	LING-R	0.37 ± 0.16	0.39 ± 0.14	0.13 (-0.46 – 0.73)	0.37
Superior occipital gyrus	left	SOG-L	0.43 ± 0.14	0.47 ± 0.16	0.27 (-0.33 – 0.86)	0.25
Superior occipital gyrus	right	SOG-R	0.42 ± 0.14	0.46 ± 0.11	0.36 (-0.24 – 0.95)	0.15
Middle occipital gyrus	left	MOG-L	0.39 ± 0.12	0.42 ± 0.16	0.17 (-0.42 – 0.77)	0.29
Middle occipital gyrus	right	MOG-R	0.35 ± 0.15	0.44 ± 0.18	0.52 (-0.09 – 1.12)	0.15
Inferior occipital gyrus	left	IOG-L	0.30 ± 0.20	0.39 ± 0.24	0.39 (-0.20 – 0.99)	0.21
Inferior occipital gyrus	right	IOG-R	0.30 ± 0.21	0.23 ± 0.25	-0.31 (-0.91 – 0.28)	0.41
Fusiform gyrus	left	FFG-L	0.33 ± 0.16	0.35 ± 0.16	0.09 (-0.51 – 0.68)	0.30
Fusiform gyrus	right	FFG-R	0.32 ± 0.14	0.36 ± 0.16	0.25 (-0.34 – 0.84)	0.22
Postcentral gyrus	left	PoCG-L	0.42 ± 0.13	0.40 ± 0.15	-0.11 (-0.70 – 0.48)	0.76
Postcentral gyrus	right	PoCG-R	0.42 ± 0.16	0.40 ± 0.14	-0.12 (-0.71 – 0.48)	0.90
Superior parietal gyrus	left	SPG-L	0.45 ± 0.13	0.49 ± 0.16	0.30 (-0.30 – 0.89)	0.15
Superior parietal gyrus	right	SPG-R	0.42 ± 0.18	0.43 ± 0.15	0.09 (-0.50 – 0.68)	0.87
Inferior parietal lobule	left	IPL-L	0.41 ± 0.12	0.45 ± 0.14	0.26 (-0.34 – 0.85)	0.20
Inferior parietal lobule	right	IPL-R	0.40 ± 0.16	0.45 ± 0.10	0.34 (-0.26 – 0.93)	0.22
Supramarginal gyrus	left	SMG-L	0.40 ± 0.14	0.45 ± 0.15	0.39 (-0.20 – 0.99)	0.18

© 2024 Ufkes S et al. *JAMA Network Open*.

Region	Hemisphere	Abbreviation	Placebo	$MgSO_4$	*g*	*p*
Supramarginal gyrus	right	SMG-R	0.35 ± 0.18	0.45 ± 0.14	0.64 (0.03 – 1.25)	0.15
Angular gyrus	left	ANG-L	0.42 ± 0.10	0.48 ± 0.17	0.43 (-0.17 – 1.03)	0.15
Angular gyrus	right	ANG-R	0.41 ± 0.12	0.43 ± 0.13	0.13 (-0.46 – 0.73)	0.41
Precuneus	left	PCUN-L	0.36 ± 0.09	0.40 ± 0.11	0.38 (-0.22 – 0.98)	0.18
Precuneus	right	PCUN-R	0.36 ± 0.11	0.39 ± 0.11	0.29 (-0.31 – 0.88)	0.28
Paracentral lobule	left	PCL-L	0.52 ± 0.14	0.52 ± 0.16	0.01 (-0.58 – 0.61)	0.76
Paracentral lobule	right	PCL-R	0.49 ± 0.15	0.48 ± 0.17	-0.06 (-0.66 – 0.53)	0.99
Caudate	left	CAU-L	0.41 ± 0.11	0.44 ± 0.15	0.21 (-0.38 – 0.81)	0.30
Caudate	right	CAU-R	0.39 ± 0.13	0.47 ± 0.16	0.56 (-0.04 – 1.16)	0.15
Putamen	left	PUT-L	0.38 ± 0.14	0.40 ± 0.13	0.13 (-0.46 – 0.73)	0.45
Putamen	right	PUT-R	0.39 ± 0.14	0.44 ± 0.15	0.29 (-0.30 – 0.89)	0.28
Pallidum	left	PAL-L	0.45 ± 0.16	0.49 ± 0.17	0.22 (-0.37 – 0.81)	0.29
Pallidum	right	PAL-R	0.48 ± 0.19	0.49 ± 0.18	0.02 (-0.57 – 0.62)	0.70
Thalamus	left	THA-L	0.36 ± 0.14	0.43 ± 0.16	0.49 (-0.11 – 1.09)	0.15
Thalamus	right	THA-R	0.37 ± 0.15	0.44 ± 0.13	0.53 (-0.07 – 1.14)	0.15
Heschl gyrus	left	HES-L	0.44 ± 0.13	0.49 ± 0.14	0.36 (-0.24 – 0.95)	0.25
Heschl gyrus	right	HES-R	0.36 ± 0.22	0.42 ± 0.22	0.28 (-0.32 – 0.87)	0.45
Superior temporal gyrus	left	STG-L	0.36 ± 0.11	0.42 ± 0.17	0.40 (-0.19 – 1.00)	0.26
Superior temporal gyrus	right	STG-R	0.32 ± 0.15	0.41 ± 0.16	0.53 (-0.08 – 1.13)	0.15
Temporal pole (superior)	left	TPOsup-L	0.30 ± 0.16	0.27 ± 0.20	-0.12 (-0.71 – 0.47)	0.84
Temporal pole (superior)	right	TPOsup-R	0.24 ± 0.16	0.32 ± 0.19	0.43 (-0.16 – 1.03)	0.30
Middle temporal gyrus	left	MTG-L	0.34 ± 0.11	0.42 ± 0.16	0.59 (-0.01 – 1.20)	0.15
Middle temporal gyrus	right	MTG-R	0.26 ± 0.11	0.40 ± 0.14	1.07 (0.44 – 1.70)	**0.003**
Temporal pole (middle)	left	TPOmid-L	0.29 ± 0.20	0.30 ± 0.21	0.02 (-0.57 – 0.61)	0.91
Temporal pole (middle)	right	TPOmid-R	0.25 ± 0.18	0.31 ± 0.20	0.30 (-0.29 – 0.90)	0.29
Inferior temporal gyrus	left	ITG-L	0.33 ± 0.18	0.34 ± 0.22	0.05 (-0.54 – 0.64)	0.59
Inferior temporal gyrus	right	ITG-R	0.34 ± 0.16	0.36 ± 0.20	0.11 (-0.48 – 0.71)	0.59
Cerebellar hemisphere	left	CBH-L	0.30 ± 0.17	0.39 ± 0.18	0.54 (-0.06 – 1.15)	0.15
Cerebellar hemisphere	right	CBH-R	0.30 ± 0.16	0.37 ± 0.21	0.41 (-0.19 – 1.00)	0.15

Treatment group values represent the mean ± standard deviation. Effect size is reported as Hedge's *g* (95% confidence interval). The *p* value is for the test that the clustering coefficient at the node differs between treatment groups, adjusted for multiple comparisons across all nodes.

© 2024 Ufkes S et al. *JAMA Network Open*.

eTable 7. Local Efficiency AUC at Each Node

Region	Hemisphere	Abbreviation	Placebo	$MgSO_4$	*g*	*p*
Precentral gyrus	left	PreCG-L	0.60 ± 0.12	0.63 ± 0.12	0.25 (-0.34 – 0.85)	0.27
Precentral gyrus	right	PreCG-R	0.57 ± 0.15	0.61 ± 0.16	0.25 (-0.35 – 0.84)	0.31
Superior frontal gyrus (dorsal)	left	SFGdor-L	0.59 ± 0.13	0.63 ± 0.11	0.32 (-0.28 – 0.91)	0.27
Superior frontal gyrus (dorsal)	right	SFGdor-R	0.59 ± 0.12	0.63 ± 0.11	0.31 (-0.29 – 0.90)	0.27
Orbitofrontal cortex (superior)	left	ORBsupb-L	0.55 ± 0.19	0.61 ± 0.14	0.36 (-0.23 – 0.96)	0.33
Orbitofrontal cortex (superior)	right	ORBsupb-R	0.57 ± 0.16	0.58 ± 0.21	0.08 (-0.51 – 0.67)	0.88
Middle frontal gyrus	left	MFG-L	0.56 ± 0.15	0.61 ± 0.11	0.38 (-0.21 – 0.98)	0.27
Middle frontal gyrus	right	MFG-R	0.59 ± 0.12	0.62 ± 0.13	0.22 (-0.38 – 0.81)	0.36
Orbitofrontal cortex (middle)	left	ORBmid-L	0.54 ± 0.19	0.60 ± 0.18	0.27 (-0.32 – 0.87)	0.40
Orbitofrontal cortex (middle)	right	ORBmid-R	0.54 ± 0.17	0.56 ± 0.24	0.08 (-0.51 – 0.67)	0.79
Inferior frontal gyrus (opercular)	left	IFGoperc-L	0.56 ± 0.16	0.62 ± 0.14	0.39 (-0.21 – 0.98)	0.27
Inferior frontal gyrus (opercular)	right	IFGoperc-R	0.59 ± 0.12	0.61 ± 0.17	0.11 (-0.48 – 0.70)	0.47
Inferior frontal gyrus (triangular)	left	IFGtriang-L	0.55 ± 0.17	0.56 ± 0.19	0.03 (-0.56 – 0.62)	0.93
Inferior frontal gyrus (triangular)	right	IFGtriang-R	0.55 ± 0.16	0.60 ± 0.17	0.27 (-0.32 – 0.86)	0.39
Orbitofrontal cortex (inferior)	left	ORBinf-L	0.52 ± 0.17	0.59 ± 0.14	0.47 (-0.13 – 1.07)	0.27
Orbitofrontal cortex (inferior)	right	ORBinf-R	0.56 ± 0.12	0.59 ± 0.16	0.23 (-0.37 – 0.82)	0.44
Rolandic operculum	left	ROL-L	0.55 ± 0.13	0.60 ± 0.16	0.31 (-0.29 – 0.90)	0.31
Rolandic operculum	right	ROL-R	0.55 ± 0.15	0.60 ± 0.17	0.30 (-0.29 – 0.90)	0.27
Supplementary motor area	left	SMA-L	0.61 ± 0.13	0.61 ± 0.19	0.03 (-0.56 – 0.62)	0.48
Supplementary motor area	right	SMA-R	0.60 ± 0.12	0.62 ± 0.18	0.08 (-0.51 – 0.67)	0.48
Olfactory	left	OLF-L	0.51 ± 0.18	0.50 ± 0.23	-0.05 (-0.64 – 0.55)	0.88
Olfactory	right	OLF-R	0.52 ± 0.19	0.53 ± 0.23	0.04 (-0.55 – 0.63)	0.88
Superior frontal gyrus (medial)	left	SFGmed-L	0.61 ± 0.13	0.64 ± 0.11	0.28 (-0.31 – 0.88)	0.28
Superior frontal gyrus (medial)	right	SFGmed-R	0.60 ± 0.13	0.63 ± 0.13	0.28 (-0.32 – 0.87)	0.35
Orbitofrontal cortex (medial)	left	ORBmed-L	0.57 ± 0.14	0.58 ± 0.19	0.11 (-0.48 – 0.70)	0.48
Orbitofrontal cortex (medial)	right	ORBmed-R	0.55 ± 0.17	0.56 ± 0.21	0.03 (-0.56 – 0.62)	0.93
Rectus gyrus	left	REC-L	0.56 ± 0.19	0.61 ± 0.17	0.24 (-0.35 – 0.84)	0.44
Rectus gyrus	right	REC-R	0.55 ± 0.20	0.62 ± 0.17	0.36 (-0.24 – 0.96)	0.33
Insula	left	INS-L	0.55 ± 0.14	0.58 ± 0.18	0.20 (-0.39 – 0.80)	0.38
Insula	right	INS-R	0.55 ± 0.13	0.61 ± 0.15	0.39 (-0.21 – 0.99)	0.27
Anterior cingulate gyrus	left	ACG-L	0.59 ± 0.12	0.60 ± 0.14	0.12 (-0.48 – 0.71)	0.58
Anterior cingulate gyrus	right	ACG-R	0.58 ± 0.13	0.61 ± 0.14	0.22 (-0.38 – 0.81)	0.31
Middle cingulate gyrus	left	MCG-L	0.58 ± 0.11	0.57 ± 0.19	-0.11 (-0.70 – 0.48)	0.78
Middle cingulate gyrus	right	MCG-R	0.58 ± 0.12	0.55 ± 0.22	-0.17 (-0.77 – 0.42)	0.95
Posterior cingulate gyrus	left	PCG-L	0.51 ± 0.17	0.58 ± 0.13	0.44 (-0.16 – 1.04)	0.27
Posterior cingulate gyrus	right	PCG-R	0.56 ± 0.10	0.57 ± 0.21	0.10 (-0.49 – 0.69)	0.55
Hippocampus	left	HIP-L	0.45 ± 0.18	0.53 ± 0.21	0.37 (-0.23 – 0.96)	0.27
Hippocampus	right	HIP-R	0.46 ± 0.16	0.53 ± 0.19	0.40 (-0.19 – 1.00)	0.27
ParaHippocampal gyrus	left	PHG-L	0.47 ± 0.16	0.48 ± 0.24	0.09 (-0.51 – 0.68)	0.75
ParaHippocampal gyrus	right	PHG-R	0.45 ± 0.19	0.51 ± 0.20	0.31 (-0.28 – 0.91)	0.27
Amygdala	left	AMYG-L	0.42 ± 0.25	0.43 ± 0.25	0.05 (-0.55 – 0.64)	0.82
Amygdala	right	AMYG-R	0.37 ± 0.24	0.50 ± 0.26	0.48 (-0.12 – 1.08)	0.27
Calcarine cortex	left	CAL-L	0.59 ± 0.11	0.63 ± 0.13	0.38 (-0.22 – 0.97)	0.27
Calcarine cortex	right	CAL-R	0.58 ± 0.12	0.62 ± 0.13	0.32 (-0.28 – 0.91)	0.27
Cuneus	left	CUN-L	0.60 ± 0.12	0.65 ± 0.11	0.46 (-0.14 – 1.06)	0.27
Cuneus	right	CUN-R	0.59 ± 0.13	0.66 ± 0.11	0.59 (-0.01 – 1.20)	0.25
Lingual gyrus	left	LING-L	0.57 ± 0.12	0.59 ± 0.16	0.12 (-0.47 – 0.72)	0.38
Lingual gyrus	right	LING-R	0.54 ± 0.17	0.59 ± 0.17	0.33 (-0.27 – 0.92)	0.27
Superior occipital gyrus	left	SOG-L	0.60 ± 0.14	0.64 ± 0.14	0.32 (-0.27 – 0.92)	0.27
Superior occipital gyrus	right	SOG-R	0.58 ± 0.14	0.65 ± 0.11	0.57 (-0.04 – 1.17)	0.25
Middle occipital gyrus	left	MOG-L	0.58 ± 0.13	0.60 ± 0.17	0.10 (-0.49 – 0.70)	0.39
Middle occipital gyrus	right	MOG-R	0.48 ± 0.17	0.58 ± 0.19	0.54 (-0.06 – 1.14)	0.26
Inferior occipital gyrus	left	IOG-L	0.38 ± 0.24	0.47 ± 0.26	0.34 (-0.26 – 0.94)	0.27
Inferior occipital gyrus	right	IOG-R	0.37 ± 0.24	0.28 ± 0.29	-0.33 (-0.93 – 0.26)	0.38
Fusiform gyrus	left	FFG-L	0.49 ± 0.19	0.49 ± 0.20	-0.02 (-0.61 – 0.57)	0.48
Fusiform gyrus	right	FFG-R	0.49 ± 0.18	0.52 ± 0.17	0.13 (-0.46 – 0.72)	0.33
Postcentral gyrus	left	PoCG-L	0.60 ± 0.12	0.60 ± 0.15	-0.03 (-0.63 – 0.56)	0.62
Postcentral gyrus	right	PoCG-R	0.58 ± 0.15	0.59 ± 0.16	0.07 (-0.52 – 0.66)	0.58
Superior parietal gyrus	left	SPG-L	0.60 ± 0.14	0.64 ± 0.16	0.27 (-0.32 – 0.87)	0.27
Superior parietal gyrus	right	SPG-R	0.56 ± 0.19	0.60 ± 0.17	0.20 (-0.39 – 0.80)	0.70
Inferior parietal lobule	left	IPL-L	0.60 ± 0.12	0.62 ± 0.15	0.18 (-0.41 – 0.78)	0.32
Inferior parietal lobule	right	IPL-R	0.54 ± 0.17	0.62 ± 0.10	0.54 (-0.06 – 1.14)	0.26
Supramarginal gyrus	left	SMG-L	0.54 ± 0.17	0.61 ± 0.17	0.38 (-0.22 – 0.98)	0.27

© 2024 Ufkes S et al. *JAMA Network Open*.

Region	Hemisphere	Abbreviation	Placebo	$MgSO_4$	*g*	*p*
Supramarginal gyrus	right	SMG-R	0.51 ± 0.21	0.61 ± 0.13	0.58 (-0.02 – 1.19)	0.27
Angular gyrus	left	ANG-L	0.58 ± 0.09	0.61 ± 0.17	0.24 (-0.36 – 0.83)	0.37
Angular gyrus	right	ANG-R	0.58 ± 0.12	0.62 ± 0.13	0.33 (-0.26 – 0.93)	0.27
Precuneus	left	PCUN-L	0.59 ± 0.12	0.64 ± 0.12	0.41 (-0.19 – 1.01)	0.26
Precuneus	right	PCUN-R	0.58 ± 0.13	0.63 ± 0.12	0.36 (-0.24 – 0.95)	0.27
Paracentral lobule	left	PCL-L	0.63 ± 0.13	0.62 ± 0.16	-0.08 (-0.67 – 0.51)	0.79
Paracentral lobule	right	PCL-R	0.62 ± 0.13	0.60 ± 0.19	-0.15 (-0.74 – 0.45)	0.96
Caudate	left	CAU-L	0.59 ± 0.12	0.60 ± 0.17	0.08 (-0.51 – 0.67)	0.57
Caudate	right	CAU-R	0.58 ± 0.13	0.64 ± 0.14	0.42 (-0.18 – 1.02)	0.27
Putamen	left	PUT-L	0.58 ± 0.14	0.60 ± 0.17	0.13 (-0.46 – 0.72)	0.44
Putamen	right	PUT-R	0.57 ± 0.14	0.62 ± 0.16	0.30 (-0.29 – 0.90)	0.27
Pallidum	left	PAL-L	0.56 ± 0.18	0.61 ± 0.17	0.27 (-0.32 – 0.87)	0.27
Pallidum	right	PAL-R	0.58 ± 0.17	0.61 ± 0.19	0.19 (-0.41 – 0.78)	0.38
Thalamus	left	THA-L	0.54 ± 0.17	0.60 ± 0.18	0.33 (-0.27 – 0.93)	0.27
Thalamus	right	THA-R	0.55 ± 0.16	0.60 ± 0.16	0.35 (-0.25 – 0.94)	0.27
Heschl gyrus	left	HES-L	0.56 ± 0.14	0.60 ± 0.15	0.23 (-0.36 – 0.83)	0.34
Heschl gyrus	right	HES-R	0.44 ± 0.25	0.51 ± 0.24	0.29 (-0.30 – 0.89)	0.47
Superior temporal gyrus	left	STG-L	0.53 ± 0.14	0.59 ± 0.19	0.32 (-0.27 – 0.92)	0.27
Superior temporal gyrus	right	STG-R	0.48 ± 0.18	0.57 ± 0.17	0.54 (-0.06 – 1.15)	0.26
Temporal pole (superior)	left	TPOsup-L	0.43 ± 0.19	0.38 ± 0.25	-0.22 (-0.82 – 0.37)	0.68
Temporal pole (superior)	right	TPOsup-R	0.36 ± 0.22	0.46 ± 0.24	0.41 (-0.19 – 1.01)	0.35
Middle temporal gyrus	left	MTG-L	0.55 ± 0.12	0.59 ± 0.17	0.23 (-0.36 – 0.82)	0.27
Middle temporal gyrus	right	MTG-R	0.44 ± 0.17	0.58 ± 0.14	0.87 (0.25 – 1.49)	**0.02**
Temporal pole (middle)	left	TPOmid-L	0.37 ± 0.22	0.38 ± 0.26	0.05 (-0.54 – 0.65)	0.88
Temporal pole (middle)	right	TPOmid-R	0.33 ± 0.23	0.38 ± 0.24	0.17 (-0.42 – 0.76)	0.48
Inferior temporal gyrus	left	ITG-L	0.45 ± 0.22	0.43 ± 0.26	-0.07 (-0.67 – 0.52)	0.88
Inferior temporal gyrus	right	ITG-R	0.48 ± 0.19	0.48 ± 0.22	0.00 (-0.59 – 0.59)	0.86
Cerebellar hemisphere	left	CBH-L	0.39 ± 0.20	0.50 ± 0.21	0.55 (-0.06 – 1.15)	0.26
Cerebellar hemisphere	right	CBH-R	0.41 ± 0.19	0.48 ± 0.24	0.32 (-0.28 – 0.91)	0.27

Treatment group values represent the mean ± standard deviation. Effect size is reported as Hedge's *g* (95% confidence interval). The *p* value is for the test that the local efficiency at the node differs between treatment groups, adjusted for multiple comparisons across all nodes.

© 2024 Ufkes S et al. *JAMA Network Open*.
