# Supplementary material for: Prenatal Magnesium Sulfate and Functional Connectivity in Offspring at Term-Equivalent Age
Source: JAMA Netw Open. 2024 May 28;7(5):e2413508. doi: 10.1001/jamanetworkopen.2024.13508 (PMC11134217; doi:10.1001/jamanetworkopen.2024.13508)
Supplement: Supplement 2. — Data Sharing Statement [file jamanetwopen-e2413508-s002.pdf]

Data Sharing Statement

Ufkes. Prenatal Magnesium Sulfate and Functional Connectivity in Offspring at Term-Equivalent Age. *JAMA Netw Open*. Published May 28, 2024.
doi:10.1001/jamanetworkopen.2024.13508

Data
Data available: Yes
Data types: Deidentified participant data
How to access data: researchhub@auckland.ac.nz
When available: With publication

Supporting Documents
Document types: None

Additional Information
Who can access the data: Data will be shared with researchers who provide a methodologically sound proposal and have appropriate ethical approval, where necessary, to achieve the research aims in the approved proposal.
Types of analyses: Use of data only for the specified proposal.
Mechanisms of data availability: Data requestors will be required to sign a Data Access Agreement before data are released. This includes a commitment to using the data only for the specified proposal, a commitment to secure storage and use of the data, and to destroy or return the data after completion of the project.
